# Antenatal Corticosteroids and Infectious Diseases Throughout Childhood

**DOI:** 10.1001/jamanetworkopen.2025.36809

**Published:** 2025-10-13

**Authors:** Fabienne Decrue, Emily M. Frier, Chun Lin, Marius Lahti-Pulkkinen, Jessica E. Miller, David Burgner, Debby Bogaert, Kate E. Duhig, Bo Jacobsson, Helga Zoega, Joshua P. Vogel, Jasper V. Been, Rebecca M. Reynolds, Sarah J. Stock

**Affiliations:** 1 Centre for Cardiovascular Science, Queen’s Medical Research Institute, University of Edinburgh, Edinburgh, United Kingdom; 2 Medical Research Council Centre for Reproductive Health, University of Edinburgh, Edinburgh, United Kingdom; 3 Department of Psychology and Logopedics, University of Helsinki, Helsinki, Finland; 4 Population Health Unit, Finnish Institute for Health and Welfare, Helsinki, Finland; 5 Murdoch Children’s Research Institute, Parkville, Victoria, Australia; 6 Department of Paediatrics, the University of Melbourne, Parkville, Victoria, Australia; 7 Center for Inflammation Research, Institute for Regeneration and Repair, University of Edinburgh, Edinburgh, United Kingdom; 8 Department of Pediatric Immunology and Infectious Diseases, University Medical Center Utrecht Wilhelmina Children’s Hospital, the Netherlands; 9 Department of Women and Children’s Health, School of Life Course and Population Sciences, King’s College London, London, United Kingdom; 10 Department of Obstetrics and Gynecology, Institute of Clinical Science, Sahlgrenska Academy, University of Gothenburg, Gothenburg, Sweden; 11 Department of Obstetrics and Gynecology, Region Västra Götaland, Sahlgrenska University Hospital, Gothenburg, Sweden; 12 Department of Genetics and Bioinformatics, Domain of Health Data and Digitalization, Institute of Public Health, Oslo, Norway; 13 School of Population Health, Faculty of Medicine and Health, University of New South Wales, Sydney, New South Wales, Australia; 14 Centre of Public Health Sciences, Faculty of Medicine, University of Iceland, Iceland; 15 Maternal, Child and Adolescent Health Program, Burnet Institute, Melbourne, Victoria, Australia; 16 Division of Neonatology, Department of Neonatal and Paediatric Intensive Care, Erasmus MC Sophia Children’s Hospital, University Medical Centre Rotterdam, Rotterdam, the Netherlands; 17 Department of Obstetrics and Gynaecology, Erasmus MC Sophia Children’s Hospital, University Medical Centre Rotterdam, Rotterdam, the Netherlands; 18 Department of Public Health, Erasmus MC Sophia Children’s Hospital, University Medical Centre Rotterdam, Rotterdam, the Netherlands; 19 Usher Institute of Population Health Sciences and Informatics, University of Edinburgh, Edinburgh, United Kingdom

## Abstract

**Importance:**

International guidelines recommend the use of antenatal corticosteroids (ACS) in pregnancies at risk of imminent preterm birth before 34 weeks’ gestation. However, whether ACS leads to long-term risk of infection from childhood to adulthood is unknown.

**Objective:**

To determine whether preterm (<37 weeks’ gestation) and full-term (37-41 weeks’ gestation) children exposed to ACS are more susceptible to respiratory and nonrespiratory infections compared with ACS-unexposed children throughout childhood and adolescence.

**Design, Setting, and Participants:**

This population-based cohort study used data from the multicenter Consortium for the Study of Pregnancy Treatments (Co-OPT) study, including data from nationwide registries for mothers and their children in Finland and Scotland. Singleton children born from 1997 to 2018 and 2006 to 2018 in Scotland and Finland, respectively, were followed up until 2018, death, or first infection. Data were analyzed between June 2022 and October 2023.

**Exposures:**

Maternal ACS treatment.

**Main Outcomes and Measures:**

Primary and secondary outcomes were the first diagnosis of respiratory or nonrespiratory infection after birth-related hospital discharge. Outcomes were stratified by gestational age at birth.

**Results:**

Among 1 548 538 included mother-child pairs (mean [SD] maternal age, 29.4 [5.7] years; mean [SD] gestational age at birth, 39.2 [1.7] weeks; 759 082 [49.0%] female neonates), 49 263 children (3.2%) were ACS-exposed, of whom 34 806 (70.7%) were preterm and 14 457 (29.3%) were full term at birth. ACS-exposed children had more respiratory and nonrespiratory infections than nonexposed children (incidence rate, 65.2 vs 39.8 and 30.0 vs 17.9 per 1000 person-years, respectively). Compared with nonexposed children, higher risks for respiratory and nonrespiratory infections were found among ACS-exposed children born at 34 weeks 0 days to 36 weeks 6 days’ gestation (adjusted hazard ratios [HRs], 1.10 [95% CI, 1.06-1.14] and 1.19 [95% CI, 1.15-1.24]), 37 0/7 to 38 6/7 weeks’ gestation (adjusted HRs, 1.27 [95% CI, 1.21-1.32] and 1.17 [95% CI, 1.11-1.23]), and 39 weeks 0 days to 41 weeks 6 days’ gestation (adjusted HRs, 1.23 [95% CI, 1.16-1.30] and 1.31 [95% CI, 1.22-1.40]). However, ACS-exposed children born at 28 weeks 0 days to 31 weeks 6 days’ gestation and 32 weeks 0 days to 33 weeks 6 days’ gestation showed no association between ACS exposure and respiratory and nonrespiratory infections.

**Conclusion and Relevance:**

In this cohort study, exposure to ACS was associated with increased risks of infections in full-term children until age 21 years. In preterm children born before 34 weeks’ gestation, no association between ACS and infections was found. To minimize the adverse effects of ACS treatment, more stringent criteria for ACS administration and better prediction tools for preterm birth are required.

## Key Points

**Question:**

Is maternal antenatal corticosteroid treatment associated with increased susceptibility for respiratory and nonrespiratory infections in offspring from childhood to adulthood?

**Findings:**

In this cohort study of 1 548 538 mother-child pairs, exposure to antenatal corticosteroid treatment was significantly associated with an increased risk of respiratory and nonrespiratory infections in preterm and full-term children through age 21 years.

**Meaning:**

These findings might help inform discussion around the benefits and long-term risks of maternal antenatal corticosteroid treatment.

## Teaser

This cohort study assesses the association between maternal antenatal corticosteroid treatment and the development of infections in offspring from childhood to adulthood.

## Introduction

Antenatal corticosteroids (ACS) are routinely administered in pregnancies between 24 weeks 0 days and 33 weeks 6 days’ gestation at risk of imminent preterm birth (<37 weeks’ gestation).^1,2^ Studies have shown better respiratory outcomes with the administration of ACS before birth at later gestations, including before late preterm birth (34 weeks 0 days to 36 weeks 6 days’ gestation),^2,3^ and before planned cesarean birth at early term gestation (37 weeks 0 days to 38 weeks 6 days’ gestation).^4^ Consequently, the proportion of children and mothers exposed to ACS has increased substantially.^5^ Approximately 50% of all children exposed to ACS are born at full term (≥37 weeks’ gestation),^6^ and a small proportion of children exposed to ACS are born within the optimal therapeutic time window (ie, within 7 days between ACS administration and birth).^7^

Animal models and cohort studies have raised concerns regarding potential associations of ACS exposure and adverse effects on cardiovascular, metabolic, respiratory, kidney, and immune and neurocognitive development.^8,9,10,11,12,13^ Whether the broad immunosuppressive mechanisms and changes in hypothalamic-pituitary-adrenal (HPA) function and lung structure resulting from ACS exposure are associated with an increased susceptibility for infectious diseases is still poorly understood. Studies have shown that ACS-exposed children have an increased short-term risk of infectious diseases during the first 4 years of life.^14,15^ However, to date, no study has investigated the long-term risk of ACS exposure on infectious diseases in childhood and adolescence through adulthood.

The aim of this study was to determine whether ACS exposure in preterm and full-term children is associated with increased susceptibility to infectious diseases throughout childhood to adulthood. To explore whether generalized vulnerability to infections is mainly driven by changes in lung structure or driven by immunosuppressive effects of ACS treatment and changes in the HPA axis, we also investigated the risks separately for respiratory and nonrespiratory infections among children who were exposed to ACS vs those who were not exposed.

## Methods

### Study Population and Period

Live-born preterm and full-term singleton children were identified for inclusion in the study from the Consortium for the Study of Pregnancy Treatments (Co-OPT) cohort.^16^ This cohort study was conducted in accordance with the Strengthening the Reporting of Observational Studies in Epidemiology (STROBE) guideline and was approved by the Public Benefit and Privacy Panel for Health and Social Care. This panel approves the use of unconsented data for research, provided the research is of public benefit; thus, informed consent was not required. The Co-OPT cohort includes prospectively obtained population-level data from Canada, Finland, Iceland, Israel, and Scotland. Pseudonymized maternity and neonatal data were linked to hospital inpatient and outpatient data, and statutory data collected information on stillbirth and child death. Long-term data on inpatient and outpatient diagnoses were not available for Canada, Iceland, and Israel. Therefore, we only included Co-OPT participants from Scotland from 1997 to 2018 and from Finland from 2006 to 2018 in this study. Scottish and Finnish participants were thus followed up until a maximum age of 21 and 12 years, respectively. Exclusion criteria were major congenital anomalies (as defined in Co-OPT cohort^16^), or missing information on ACS exposure, either because of missing ACS data or ACS coded as unknown (eFigure 1 in Supplement 1). Further, children from the Co-OPT cohort born before 28 weeks 0 days’ gestation and after 42 weeks 0 days’ gestation were not included in this study.

### Exposures

Children were classified as exposed if they were born to mothers who received at least 1 dose of ACS. This information was recorded in the Scottish Maternity Inpatient and Day Case dataset and the Finnish Medical Birth Register.

### Follow-Up and Outcomes

Primary and secondary outcomes were the first diagnosis of respiratory infection and first diagnosis of nonrespiratory infection, respectively. All infectious disease outcomes were coded according to the *International Statistical Classification of Disease and Related Health Problems*, *Tenth Revision* (*ICD-10*).^17^ Data on infections were extracted from Scottish Morbidity Records, which included episode-level data on inpatient and day case records, and from the Finnish Care Register for Health Care, which included episode-level data on inpatient and outpatient data. Primary and all secondary diagnostic codes were searched for *ICD-10* codes of infections. *ICD-10* codes were grouped into 7 clinical categories in accordance with previous literature (list of *ICD-10* codes in Supplement 1).^18^

Respiratory infections were defined as either lower respiratory tract infections (LRTIs) or upper respiratory tract infections (URTIs). Nonrespiratory infection subgroups included invasive bacterial, gastrointestinal, skin and soft tissue, genitourinary, or viral infections (no other specified viral causes).

### Covariates

Covariates were chosen based on their association with exposure and outcome based on literature review and based on a direct acyclic graph (DAG) (eFigure 2 in Supplement 1). Various potential covariates from the DAG could not be included as no data were available for these factors or due to a high percentage of missing information.^16^ The final model included maternal age at birth (in years), parity, maternal smoking at first antenatal appointment or first trimester, maternal diabetes, gestational age at birth (in weeks), country (Scotland or Finland), mode of birth (cesarean or vaginal), year of birth, sex of child, and birth weight centiles (adjusted for sex and gestational age^19^). More detailed information on covariates are given in eMethods in Supplement 1.

### Statistical Analyses

For all outcomes, ACS-exposed children were compared with children not exposed to ACS (nonexposed children). Outcomes for exposure and comparison groups were stratified based on gestational age categories at birth (completed weeks at birth) according to World Health Organization definitions.^20^ However, moderate-to-late preterm children were divided into 2 groups, as the clear benefit of ACS treatment until 33 weeks’ gestation has been shown,^1,2^ whereas the long-term benefit-risk ratio for children born at 34 weeks 0 days to 36 weeks 6 days’ gestation is still unknown. Therefore, we created subgroups for children born very preterm (28 weeks 0 days to 31 weeks 6 days’ gestation), moderate preterm (32 weeks 0 days to 33 weeks 6 days’ gestation), late preterm (34 weeks 0 days to 36 weeks 6 days’ gestation), early term (37 weeks 0 days to 38 weeks 6 days’ gestation), and late or full term (39 weeks 0 days to 41 weeks 6 days’ gestation).

For each of the infectious disease subgroups, we calculated the incidence rate of first infection per 1000 person-years for each gestational group. Cox proportional hazards regression was used to estimate hazard ratio (HR) with 95% CI for first respiratory or nonrespiratory infection (any type of either infection) among ACS-exposed vs nonexposed children, with child’s age as the time scale. Participants were followed up from discharge from birth-related hospitalization until first respiratory or nonrespiratory infection, end of follow-up, or death, whichever occurred first. We used a simple adjusted model in the first step (adjusted for child’s sex and year of birth) and in a second step adjusted for all covariates listed above. Cox proportionality assumptions were tested by using Kaplan-Meier survival curves. *P* < .05 was considered statistically significant. Statistical analyses were conducted between June 2022 and October 2023 using STATA, version 16.1 (StataCorp LLC) and R, version 4.2.0 (R Core Team).

To account for missing data in the Scottish Maternity Inpatient and Day Case dataset and the Finnish Medical Birth Register,^16^ we performed a sensitivity analysis including children with a full set of all covariates only. Further, we performed stratified sensitivity analyses to check for discrepancies between countries and dependence of age.

## Results

### Study Population and Characteristics

A total of 1 929 157 mother-child pairs were identified in the Scottish and Finnish registries, from which 1 548 538 (80.3%) were included in the study (mean [SD] maternal age, 29.4 [5.7] years; mean [SD] gestational age at birth, 39.2 [1.7] weeks; 759 082 [49.0%] female neonates) (eFigure 1 and eTable 1 in Supplement 1). Of these, 887 290 participants (57.3%) were Scottish and 661 248 (42.7%) were Finnish. Overall, 49 263 children (3.2%) were exposed to ACS, from which 34 806 (70.7%) were born preterm and 14 457 (29.3%) born full term. Anthropometric data and covariates are shown in Table 1 and stratified by country, term birth, and ACS exposure in eTables 2-4 in Supplement 1. Median age at first episode of infectious disease was 494 days (IQR, 215-1070 days) for respiratory and 567 days (245-1281 days) for nonrespiratory infectious disease (eTable 5 in Supplement 1). Differences between ACS-exposed and nonexposed participants are shown (Table 1; eTable 4 in Supplement 1).

**Table 1.** Descriptive Characteristics of Study Population

Characteristic	Combined (N = 1 548 538)^a^		ACS^a^			
			Exposed (n = 49 263)		Nonexposed (n = 1 499 275)	
	No. (%)	Missing, No. (%)	No. (%)	Missing, No. (%)	No. (%)	Missing, No. (%)
Preterm (gestational age 28 wk 0 d to 36 wk 6 d)	86 482 (5.58)	0	34 806 (70.65)	0	51 676 (3.45)	0
Full term (gestational age 37 wk 0 d to 41 wk 6 d)	1 462 056 (94.42)	0	14 457 (29.35)	0	1 447 599 (96.55)	0
Maternal age, mean (SD), y	29.4 (5.70)	<5	29.75 (6.06)	0	29.39 (5.70)	<5
Maternal BMI, mean (SD)	25.2 (5.30)	299 061 (19.3)	25.76 (5.99)	9775 (19.8)	25.24 (5.32)	289 286 (19.3)
Parity						
0	653 672 (42.20)	5761 (0.4)	22 175 (45.01)	275 (0.6)	631 487 (42.12)	5486 (0.4)
1	537 323 (34.70)		14 685 (29.81)		522 638 (34.86)	
2	221 177 (14.30)		6983 (14.17)		214 194 (14.29)	
≥3	124 844 (8.10)		5420 (11.00)		130 946 (8.73)	
Smoking	278 762 (18.00)	56 678 (3.7)	11 153 (22.64)	2586 (5.2)	267 609 (17.85)	54 092 (3.6)
Any diabetes	98 177 (6.34)	105 920 (6.8)	3230 (6.56)	28 926 (58.7)	80 283 (5.35)	829 139 (55.3)
Gestational diabetes	83 513 (5.39)	858 065 (55.4)	73 706 (11.15)	0	9807 (1.11)	858 065 (57.2)
Hypertension	8499 (0.55)	864 421 (55.8)	735 (1.49)	29 274 (59.4)	7764 (0.52)	835 147 (55.7)
Preeclampsia	62 275 (4.02)	838 511 (54.1)	5234 (10.62)	27 056 (54.9)	57 041 (3.80)	811 455 (54.1)
Cesarean birth	338 741 (21.87)	<5	25 119 (50.99)	0	313 622 (20.92)	<5
Birth weight, mean (SD), g	3443.9 (551.4)	1442 (0.1)	2456.3 (789.7)	162 (0.3)	3476.3 (510.4)	1280 (0.1)
Sex at birth						
Female	759 082 (49.02)	95 (<0.1)	23 144 (46.98)	10 (<0.1)	735 938 (49.09)	85 (<0.1)
Male	789 456 (50.98)		21 119 (53.02)		763 337 (50.91)	
Apgar score <7^b^	24 490 (1.58)	92 862 (6.0)	3128 (6.35)	4001 (8.1)	21 362 (1.42)	88 861 (5.9)
NICU admission	143 633 (9.28)	14 916 (1.0)	27 690 (56.21)	925 (1.9)	115 943 (7.73)	13 991 (0.9)
Gestational age at birth, mean (SD), wk	39.2 (1.70)	0	34.77 (3.02)	0	39.37 (1.39)	0

Abbreviations: ACS, antenatal corticosteroid; BMI, body mass index (calculated as weight in kilograms divided by height in meters squared); NICU, neonatal intensive care unit.

^a^ Data provided for population included in the study after exclusion criteria (missing information on ACS and other major congenital anomalies) were applied.

^b^ An Apgar score of less than 7 indicates that an infant might need immediate medical attention.

### Incidence Rates of First Infectious Diseases

The absolute numbers and incidence rates of first infection by infectious subgroup (ie, URTI, LRTI, gastrointestinal, genitourinary, skin and soft tissue, bacterial, and viral) are shown in Table 2. Throughout childhood to adulthood, ACS-exposed children had more inpatient and outpatient diagnoses with respiratory and nonrespiratory infections (incidence rate, 65.2 and 30.0 per 1000 person-years) than nonexposed children (incidence rate, 39.8 vs 17.9 per 1000 person-years). Absolute numbers and incidence rates of first infection divided into infectious subgroups and further divided by gestational age at birth are shown in Table 3. Incidence rates for first respiratory and first nonrespiratory infections decreased with increasing gestational age at birth. Children born at 28 weeks 0 days to 31 weeks 6 days’ gestation showed the highest incidence rates of respiratory and nonrespiratory infections among all children at 82 and 32 per 1000 person-years, respectively. However, children born at 39 weeks 0 days to 41 weeks 6 days’ gestation showed the lowest incidence rates for respiratory and nonrespiratory infections at 38.2 and 17.3 per 1000 person-years, respectively.

**Table 2.** Absolute Number and Incidence Rate (IR) of First Infectious Disease Throughout Childhood and Adolescence

Category	Combined			ACS					
				Exposed			Nonexposed		
	No. (%)	PY	IR per 1000 PY	No. (%)	PY	IR per 1000 PY	No. (%)	PY	IR per 1000 PY
Respiratory infections	414 480 (26.8)	10 248 578	40.4	16 940 (34.4)	259 918	65.2	397 540 (26.5)	9 988 659	39.8
URTI	330 817 (21.4)	10 856 616	30.5	12 212 (24.8)	291 793	41.9	318 605 (21.3)	10 564 822	30.2
LRTI	157 800 (10.2)	11 731 379	13.5	8599 (17.5)	305 479	28.1	149 201 (10.0)	10 564 822	30.2
Nonrespiratory infections	208 946 (13.5)	11 439 383	18.3	9152 (18.6)	305 296	30.0	199 749 (13.3)	11 134 087	17.9
Gastrointestinal	56 189 (3.6)	12 361 491	4.5	2447 (5.0)	343 212	7.1	53 741 (3.6)	12 018 279	4.5
Skin and soft tissue	23 026 (1.5)	12 635 114	1.8	757 (1.5)	354 232	2.1	22 269 (1.5)	12 280 882	1.8
Genitourinary	26 205 (1.7)	12 604 726	2.1	1130 (2.3)	351 344	3.2	25 074 (1.7)	12 253 382	2.0
Invasive bacterial	14 075 (0.9)	12 659 301	1.1	640 (1.3)	353 850	1.8	13 392 (0.9)	12 305 451	1.1
Viral	116 741 (7.5)	12 060 526	9.7	5729 (11.6)	327 077	17.5	111 012 (7.5)	11 733 448	9.5

Abbreviations: ACS, antenatal corticosteroid; LRTI, lower respiratory tract infections; PY, person-years; URTI, upper respiratory tract infections.

**Table 3.** Absolute Number and Incidence Rate (IR) of First Infectious Disease Divided by Gestational Age

Category	Gestational age group														
	28 wk 0 d to 31 wk 6 d			32 wk 0 d to 33 wk 6 d			34 wk 0 d to 36 wk 6 d			37 wk 0 d to 38 wk 6 d			39 wk 0 d to 41 wk 6 d		
	No. (%)	PY	IR per 1000 PY	No. (%)	PY	IR per 1000 PY	No. (%)	PY	IR per 1000 PY	No. (%)	PY	IR per 1000 PY	No. (%)	PY	IR per 1000 PY
Respiratory infections	3966 (45.4)	48 404	81.93	4118 (37.3)	67 781	60.75	21 411 (32.1)	413 038	51.84	83 478 (29.0)	1 835 773	45.47	301 507 (25.7)	7 883 581	38.24
URTI	2751 (31.5)	58 316	45.44	2962 (26.8)	76 910	38.51	16 198 (24.3)	451 739	35.86	65 834 (22.9)	1 963 146	33.53	243 072 (20.7)	8 306 504	29.26
LRTI	2423 (27.7)	58 782	41.22	2182 (19.8)	79 826	27.33	9775 (14.7)	482 493	20.26	33 789 (11.8)	2 125 255	15.90	109 631 (9.3)	8 985 023	12.20
Nonrespiratory infections	2032 (23.3)	62 712	32.39	2113 (19.2)	80 859	26.13	11 042 (16.6)	477 295	23.13	42 572 (14.8)	2 076 434	20.50	151 187 (12.9)	8 742 083	17.29
Gastrointestinal	496 (5.7)	72 838	6.81	571 (5.2)	90 984	6.28	3047 (4.6)	526 792	5.78	11 818 (4.1)	2 260 351	5.23	40 257 (3.4)	9 410 527	4.28
Skin and soft tissue	160 (1.8)	75 402	2.12	173 (1.6)	93 829	1.84	1025 (1.5)	542 264	1.89	4550 (1.6)	2 318 405	1.96	17 118 (1.5)	9 605 215	1.78
Genitourinary	296 (3.4)	74 318	3.97	288 (2.6)	92 831	3.10	1353 (2.0)	539 536	2.51	5260 (1.8)	2 311 884	2.28	19 008 (1.6)	9 586 157	1.98
Invasive bacterial	167 (1.9)	75 097	2.22	143 (1.3)	93 915	1.52	817 (1.2)	541 962	1.51	2684 (0.9)	2 323 620	1.15	10 264 (0.9)	9 624 718	1.06
Viral	1330 (15.2)	67 959	18.55	1287 (11.7)	86 852	14.03	6604 (9.9)	508 053	13.00	24 322 (8.5)	2 199 182	10.92	83 198 (7.1)	9 198 481	9.04

Abbreviations: LRTI, lower respiratory tract infections; PY, person-years; URTI, upper respiratory tract infections.

### Association of ACS Exposure and Respiratory Infectious Diseases

We estimated the association of ACS exposure and risk of infection throughout childhood and adolescence for children by gestational age group (Table 4; Figure). Overall, ACS-exposed children showed increased risk of respiratory infection (HR, 1.19; 95% CI, 1.16-1.21) compared with those who were not exposed.

**Table 4.** Association of Exposure to Antenatal Corticosteroids With Infectious Diseases Throughout Childhood

Category	No.				HR (95% CI)	
	Exposed, total No.	Exposed, cases	Unexposed, total No.	Unexposed, cases	Simple adjusted^a^	Full adjusted^b^
**Respiratory infections by gestational age group, wk**						
28 wk 0 d to 31 wk 6 d	7186	3242	1553	724	0.95 (0.88-1.03)	1.06 (0.98-1.17)
32 wk 0 d to 33 wk 6 d	8277	2941	2758	1177	0.81 (0.76-0.87)	1.05 (0.97-1.13)
34 wk 0 d to 36 wk 6 d	19 343	5966	47 365	15 445	0.98 (0.95-1.01)	1.10 (1.06-1.14)
37 wk 0 d to 38 wk 6 d	8829	2700	278 745	80 778	1.18 (1.12-1.22)	1.19 (1.15-1.24)
39 wk 0 d to 41 wk 6 d	5628	2091	1 168 854	299 416	1.62 (1.55-1.69)	1.27 (1.21-1.32)
**Nonrespiratory infections by gestational age group, wk**						
28 wk 0 d to 31 wk 6 d	7186	1700	1553	332	1.14 (1.02-1.29)	0.97 (0.85-1.10)
32 wk 0 d to 33 wk 6 d	8277	1644	2758	469	1.23 (1.11-1.37)	0.99 (0.88-1.11)
34 wk 0 d to 36 wk 6 d	19 343	3510	47 365	7532	1.24 (1.19-1.29)	1.17 (1.11-1.23)
37 wk 0 d to 38 wk 6 d	8829	1418	278 745	41 154	1.28 (1.21-1.35)	1.23 (1.16-1.30)
39 wk 0 d to 41 wk 6 d	5628	881	1 168 854	150 306	1.30 (1.22-1.39)	1.31 (1.22-1.40)

Abbreviations: ACS, antenatal corticosteroids; HR, hazard ratio.

^a^ Simple adjusted model was adjusted for sex and year of birth.

^b^ Full adjusted model was adjusted for sex, year of birth, gestational age at birth, country, mode of delivery, maternal age, parity, maternal smoking, maternal diabetes, and birth weight; birth weight was adjusted for gestational age and sex.

**Figure.**
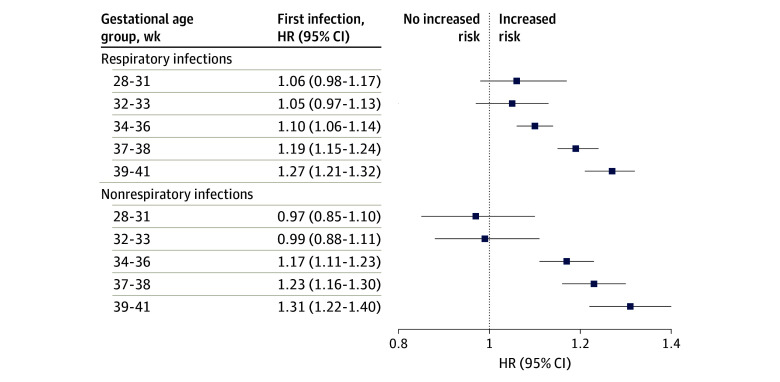
Hazard Ratios (HRs) for Respiratory and Nonrespiratory Infections by Gestational Age Forest plot showing adjusted HRs for respiratory and nonrespiratory infections stratified by gestational age group calculated from Cox proportional hazards models. Full models were adjusted for sex, year of birth, gestational age at birth, country, mode of delivery, maternal age, parity, maternal smoking, maternal diabetes, and birth weight; birth weight was adjusted for gestational age and sex.

Upon stratification, we observed that ACS-exposed children born at 28 weeks 0 days to 31 weeks 6 days’ gestation (HR, 1.06; 95% CI, 0.98-1.17) and 32 weeks to 33 weeks’ gestation (HR, 1.05; 95% CI, 0.97-1.13) showed no association between ACS exposure and respiratory infections in the full adjusted model. ACS-exposed children born at 34 weeks 0 days to 36 weeks 6 days’ gestation showed no association in the simple adjusted model (adjusted for sex and year of birth) (HR, 0.98; 95% CI, 0.95-1.01); however, they showed increased risk of respiratory infection in the full adjusted model (HR, 1.10; 95% CI, 1.06-1.14). ACS-exposed children born at 37 weeks 0 days to 38 weeks 6 days’ gestation and 39 weeks 0 days to 41 weeks 6 days’ gestation showed significantly increased risk of respiratory infection in the full adjusted models (HR, 1.19; 95% CI, 1.15-1.24 and HR, 1.27; 95% CI, 1.21-1.32, respectively).

### Association of ACS Exposure and Nonrespiratory Infectious Diseases

Risk of nonrespiratory infection throughout childhood was higher among ACS-exposed compared with nonexposed children in the overall population (HR, 1.15; 95% CI, 1.12-1.18) and in the stratified analyses by gestational age groups. Children born at 28 weeks 0 days to 31 weeks 6 days’ gestation and 32 weeks 0 days to 33 weeks 6 days’ gestation had increased risk of nonrespiratory infection in the simple adjusted model (HR, 1.14; 95% CI, 1.02-1.29; and HR, 1.23; 95% CI, 1.11-1.37, respectively). However, in the full adjusted model, ACS exposure was not associated with nonrespiratory infections among children born at 28 weeks 0 days to 31 weeks 6 days’ gestation (HR, 0.97; 95% CI, 0.85-1.10) nor those born at 32 weeks 0 days to 33 weeks 6 days’ gestation (HR, 0.99; 95% CI, 0.88-1.11).

The simple and full adjusted models showed significantly increased risks for nonrespiratory infectious diseases among ACS-exposed children born at 34 weeks 0 days to 36 weeks 6 days’ gestation, 37 weeks 0 days to 38 weeks 6 days’ gestation, and 39 weeks 0 days to 41 weeks 6 days’ gestation. In the full adjusted model, the HRs for these gestational age groups were 1.17 (95% CI, 1.11-1.23), 1.23 (95% CI, 1.16-1.30), and 1.31 (95% CI, 1.22-1.40), respectively. eFigures 3-12 in Supplement 1 depict the Kaplan-Meier curves of respiratory and nonrespiratory infections for each gestational age group.

### Sensitivity Analyses

When we included only children with full information on all covariates in the regression model, including maternal body mass index (BMI), maternal hypertension, and preeclampsia, the number of participants with available data on all covariates decreased (eTable 6 in Supplement 1). However, the results with or without inclusion of these covariates did not differ from the findings in our main analyses. In the stratified analyses by country, there were similar findings to our main analyses (eTable 7 in Supplement 1). Analyses examining HRs across child age are shown in eTable 8 in Supplement 1.

## Discussion

In this large population-based cohort study from Finland and Scotland, we included a total of 1 548 538 mother-child pairs and followed them up to a maximum age of 21 years. Overall, 49 263 children from the whole cohort were exposed to ACS. Notably, this is the first study investigating the long-term associations of ACS exposure on infections throughout childhood until adulthood. Previous investigations on ACS exposure have mainly focused on neonatal benefits and risks of ACS exposure, such as reduced neonatal mortality, respiratory distress syndrome, or neonatal sepsis.^1,2,3,4^ Few studies have examined the association of ACS exposure and infections in the first months of life.^14^ Our study showed an increased risk of infection throughout childhood to adulthood (maximum age of 21 years) among ACS-exposed children.

We found the incidence rate of first infection was inversely correlated with gestational age at birth, in which very early preterm children (28 weeks 0 days to 31 weeks 6 days’ gestation) showed the highest incidence rates per 1000 person-years. This finding is consistent with previous findings from an English population-based linkage study^21^ that showed gestational age was negatively associated with infection-related hospitalizations among children from birth to age 10 years. The decline in incidence rate with increasing gestational age could largely be explained by the pronounced immaturity of the innate and adaptive immune system in preterm born children compared with their full-term peers,^22^ which is also evident in school-aged preterm children.^23^

ACS-exposed preterm and full-term children were at increased risk of respiratory infection throughout childhood and adolescence when compared with their nonexposed peers. This finding is supported by previously published findings from a population-based study in Taiwan by Yao et al.^14^ In this study, the authors investigated the association of ACS exposure and severe infections throughout the first year of life. The study showed that full-term children had increased risks for pneumonia during the first 3 to 12 months of life. However, in the preterm population, ACS exposure showed no association with pneumonia during the first 3 months but was significantly associated with increased risk of pneumonia after 3 months to 12 months. In contrast to our study, Yao et al^14^ used hospital admissions only, and no outpatient data were included. Additionally, the study only included pneumonia diagnoses in analyses, without other respiratory infection diagnoses. These 2 differences, combined with the shorter follow-up of 12 months, might have accounted for differences in incidence rates and HRs when compared with our findings.

The risk of nonrespiratory infection, defined as invasive bacterial, gastrointestinal, skin and soft tissue, genitourinary, or viral infections, was also increased among ACS-exposed preterm and full-term children. Underlying biological mechanisms can be postulated and might include alteration of the HPA axis, the microbiome, and immune responses. This finding is consistent with a population-based cohort study from Finland.^15^ Räikkönen and colleagues^15^ examined the risk of infectious disease diagnoses after ACS exposure in approximately 850 000 children. Both preterm and full-term children exposed to ACS showed more inpatient treatment days, inpatient treatment episodes, and specialty care outpatient visits with infectious disease diagnoses between 0 and 4 years of age than nonexposed children. Notably, *ICD-10* coding and adjustment for covariates between our study and the Finnish study differed slightly, but results showed the same associations.

Very preterm (28 weeks 0 days to 31 weeks 6 days’ gestation) and moderate preterm (32 weeks 0 days to 33 weeks 6 days’ gestation) children showed no associations between ACS exposure and risk of respiratory nor nonrespiratory infection in the full adjusted models. In these populations, short-term benefits of ACS treatment outweighed other short-term to long-term risks. Therefore, our results illustrate the potential safety of ACS treatment in children born before 34 weeks’ gestation. However, children born at 28 weeks 0 days to 31 weeks 6 days’ gestation and 32 weeks 0 days to 33 weeks 6 days’ gestation went from an increased risk of nonrespiratory infection in the simple adjusted model to no association in the full adjusted model. These differences might be driven by the inclusion of major covariates, such as maternal smoking, maternal age, and year of birth, in the full adjusted model. Previous findings on the negative associations of maternal smoking during pregnancy or maternal age and increased risk of infection during childhood have been shown widely.^24,25^ The year of birth might have influenced our findings because ACS administration practice has changed during the study period of 21 years, with possible variation in treatment (eg, introduction of respiratory syncytial virus vaccination).^26^ These findings are further in agreement with the Finnish study by Räikkönen et al^15^ that showed no association between ACS exposure and infections in very-to-moderate preterm children (<34 weeks’ gestation) up to the age of 4 years.

The effects that ACS have on the immune system and HPA function could be the underlying mechanisms of increased susceptibility to infections. ACS administration results in changes in immune function, with a reduced capacity of T-cells to proliferate, decreased absolute lymphocyte count, and reduced CD4+ and CD25+ lymphocytes in cord blood samples.^27^ In animal models, ACS injection to pregnant mice led to atrophic lymphoid tissues, adrenals, and thymus,^28^ which was partially shown in later studies in humans with reduced thymus size in the offspring after ACS exposure.^9^

Whether ACS treatment affects the fetal HPA axis depends on the maturation stage of glucocorticoid receptor expression in the fetal HPA axis or those regions involved in its regulation.^29^ ACS administration results in suppressed cortisol levels in preterm infants and increased HPA function with heightened stress response in full-term infants and children.^27^ It is unknown whether these changes in HPA function and cortisol levels resulting from ACS exposure are the causes for an increased susceptibility for infections.

### Limitations

A limitation of our study was the high level of missing data for specific covariates^16^ in the Scottish Maternity Inpatient and Day Case dataset and the Finnish Medical Birth Register, respectively. These covariates included maternal socioeconomic status, maternal hypertension, maternal BMI, marital status during pregnancy, maternal and child ethnicity, use of antenatal antibiotics, and time-varying variables, such as child care (ie, nursery attendance) or smoking status of the child at later age. Due to missingness, these variables were not included in the main analyses, which might have biased the findings in different directions. For example, we could speculate that children born to mothers with comorbidities or lower socioeconomic status might be more susceptible to infections after ACS exposure, whereas use of antenatal antibiotics might be protective. Nevertheless, the results from the sensitivity analyses, which only included children with full information on all covariates (eTable 5 in Supplement 1), showed no significant differences compared with the findings from our main analyses. Information on dosage, indication, and gestational age at administration of corticosteroids was also missing. Therefore, dose- or time-dependent effects could not be assessed. Due to lack of information on ethnicity, we cannot examine the generalizability of the results to other populations. Further studies are needed in different populations.

## Conclusions

In this prospective, population-based cohort study from Finland and Scotland, exposure to ACS was associated with increased risk of infection throughout childhood to adulthood in preterm and full-term children to the age of 21 years. Overall, our findings suggest that ACS treatment should be used judiciously, given the potential long-term effects in otherwise healthy full-term and preterm children. Mechanistic studies are warranted to identify how ACS affect susceptibility to infections and to allow development of interventions. In addition, to reduce the potential adverse consequences of ACS, more stringent criteria for ACS administration, weighing risks vs benefits in the short, and long term, and better prediction tools for preterm birth are required. This information would facilitate more targeted administration of ACS to women most likely to deliver very preterm or preterm and whose offspring are most likely to benefit.

## Notes

Supplement 1.
eMethods
**eTable 1.** Number of excluded children **eTable 2.** Descriptive characteristics according to country **eTable 3.** Descriptive characteristics according to term birth **eTable 4.** Descriptive characteristics according to ACS exposure **eTable 5.** Median age at first infection and median age at end of follow-up **eTable 6.** Sensitivity analyses including maternal BMI or hypertension or preeclampsia diagnoses into full adjusted model **eTable 7.** Sensitivity analyses performing stratified analyses by country **eTable 8.** Sensitivity analyses analysing the association of exposure to antenatal corticosteroids and infectious diseases throughout different ages of childhood and adolescence **eFigure 1.** Flowchart of exclusion criteria **eFigure 2.** Complex direct acyclic graph (DAG) **eFigure 3.** Kaplan-Meier curve of respiratory infections for preterm children born 28^+0^ – 31^+6^ weeks gestation **eFigure 4.** Kaplan-Meier curve of respiratory infections for preterm children born 32^+0^ – 33^+6^ weeks gestation **eFigure 5.** Kaplan-Meier curve of respiratory infections for preterm children born 34^+0^ – 36^+6^ weeks gestation **eFigure 6.** Kaplan-Meier curve of respiratory infections for preterm children born 37^+0^ – 38^+6^ weeks gestation **eFigure 7.** Kaplan-Meier curve of respiratory infections for term children born 39^+0^ – 41^+6^ weeks gestation **eFigure 8.** Kaplan-Meier curves of non-respiratory infections for preterm children born 28^+0^ – 31^+6^ weeks gestation **eFigure 9.** Kaplan-Meier curves of non-respiratory infections for preterm children born 32^+0^ – 33^+6^ weeks gestation **eFigure 10.** Kaplan-Meier curves of non-respiratory infections for preterm children born 34^+0^ – 36^+6^ weeks gestation **eFigure 11.** Kaplan-Meier curves of non-respiratory infections for preterm children born 37^+0^ – 38^+6^ weeks gestation **eFigure 12.** Kaplan-Meier curves of non-respiratory infections for preterm children born 39^+0^ – 41^+6^ weeks gestation

Supplement 2.
Consortium for the Study of Pregnancy Treatments (Co-OPT) Investigators

Supplement 3.
Data Sharing Statement
