# Supplementary material for: Antenatal Corticosteroids and Infectious Diseases Throughout Childhood
Source: JAMA Netw Open. 2025 Oct 13;8(10):e2536809. doi: 10.1001/jamanetworkopen.2025.36809 (PMC12519305; doi:10.1001/jamanetworkopen.2025.36809)
Supplement: Supplement 1. — eMethods eTable 1. Number of excluded children eTable 2. Descriptive characteristics according to country eTable 3. Descriptive characteristics according to term birth eTable 4. Descriptive characteristics according to ACS exposure eTable 5. Median age at first infection and median age at end of follow-up eTable 6. Sensitivity analyses including maternal BMI or hypertension or preeclampsia diagnoses into full adjusted model eTable 7. Sensitivity analyses performing stratified analyses by country eTable 8. Sensitivity analyses analysing the association of exposure to antenatal corticosteroids and infectious diseases throughout different ages of childhood and adolescence eFigure 1. Flowchart of exclusion criteria eFigure 2. Complex direct acyclic graph (DAG) eFigure 3. Kaplan-Meier curve of respiratory infections for preterm children born 28+0 – 31+6 weeks gestation eFigure 4. Kaplan-Meier curve of respiratory infections for preterm children born 32+0 – 33+6 weeks gestation eFigure 5. Kaplan-Meier curve of respiratory infections for preterm children born 34+0 – 36+6 weeks gestation eFigure 6. Kaplan-Meier curve of respiratory infections for preterm children born 37+0 – 38+6 weeks gestation eFigure 7. Kaplan-Meier curve of respiratory infections for term children born 39+0 – 41+6 weeks gestation eFigure 8. Kaplan-Meier curves of non-respiratory infections for preterm children born 28+0 – 31+6 weeks gestation eFigure 9. Kaplan-Meier curves of non-respiratory infections for preterm children born 32+0 – 33+6 weeks gestation eFigure 10. Kaplan-Meier curves of non-respiratory infections for preterm children born 34+0 – 36+6 weeks gestation eFigure 11. Kaplan-Meier curves of non-respiratory infections for preterm children born 37+0 – 38+6 weeks gestation eFigure 12. Kaplan-Meier curves of non-respiratory infections for preterm children born 39+0 – 41+6 weeks gestation [file jamanetwopen-e2536809-s001.pdf]

Supplemental Online Content

Decrue F, Frier EM, Lin C, et al. Antenatal corticosteroids and infectious diseases throughout childhood. *JAMA Netw Open.* 2025;8(10):e2536809.
doi:10.1001/jamanetworkopen.2025.36809

eMethods
eTable 1. Number of excluded children
eTable 2. Descriptive characteristics according to country
eTable 3. Descriptive characteristics according to term birth
eTable 4. Descriptive characteristics according to ACS exposure
eTable 5. Median age at first infection and median age at end of follow-up
eTable 6. Sensitivity analyses including maternal BMI or hypertension or preeclampsia diagnoses into full adjusted model
eTable 7. Sensitivity analyses performing stratified analyses by country
eTable 8. Sensitivity analyses analysing the association of exposure to antenatal corticosteroids and infectious diseases throughout different ages of childhood and adolescence
eFigure 1. Flowchart of exclusion criteria
eFigure 2. Complex direct acyclic graph (DAG)
eFigure 3. Kaplan-Meier curve of respiratory infections for preterm children born 28^{+0} – 31^{+6} weeks gestation
eFigure 4. Kaplan-Meier curve of respiratory infections for preterm children born 32^{+0} – 33^{+6} weeks gestation
eFigure 5. Kaplan-Meier curve of respiratory infections for preterm children born 34^{+0} – 36^{+6} weeks gestation
eFigure 6. Kaplan-Meier curve of respiratory infections for preterm children born 37^{+0} – 38^{+6} weeks gestation
eFigure 7. Kaplan-Meier curve of respiratory infections for term children born 39^{+0} – 41^{+6} weeks gestation
eFigure 8. Kaplan-Meier curves of non-respiratory infections for preterm children born 28^{+0} – 31^{+6} weeks gestation
eFigure 9. Kaplan-Meier curves of non-respiratory infections for preterm children born 32^{+0} – 33^{+6} weeks gestation
eFigure 10. Kaplan-Meier curves of non-respiratory infections for preterm children born 34^{+0} – $36^{+}6$ weeks gestation
eFigure 11. Kaplan-Meier curves of non-respiratory infections for preterm children born 37^{+0} – 38^{+6} weeks gestation
eFigure 12. Kaplan-Meier curves of non-respiratory infections for preterm children born 39^{+0} – 41^{+6} weeks gestation

This supplemental material has been provided by the authors to give readers additional information about their work.

eMethods

Study Population and Period

Live-born preterm and term born singleton children, born between 28^{+0} and 41^{+6} weeks gestation, were identified for inclusion in the study from the Consortium for the Study of Pregnancy Treatments (Co-OPT) cohort.[1] The Co-OPT cohort includes prospectively obtained data on a population level from Canada, Finland, Iceland, Israel, and Scotland. Pseudonymised maternity and neonatal data were linked to hospital in- and outpatient data and statutory collected information on stillbirth and child death. Long-term data on in- and outpatient diagnoses were not available for Canada, Iceland, and Israel. Therefore, we only included Co-OPT participants from Scotland from 1997 to 2018 and from Finland from 2006 to 2018 in this study. Scottish and Finnish participants were thus followed up until a maximum of 21 and 12 years of age, respectively. Exclusion criteria were major congenital anomalies (as defined in Co-OPT cohort[1]), or missing information on ACS exposure, either because of missing ACS data or ACS coded as "Unknown" (Figure E2). Further, children from the Co-OPT cohort born before 28^{+0} and after 42^{+0} weeks gestation were not included in this study. Children in these gestation groups were mostly ACS-exposed (<28 weeks' gestation) or non-exposed (≥42 weeks' gestation), leaving only small numbers of patients with or without infectious diseases in the opposite group for comparison.

Exposures

Children were classified as exposed if they were born to mothers who received at least one dose of ACS. This information was recorded in the Scottish Maternity Inpatient and Day Case dataset (SMR02) and the Finnish Medical Birth Register.

Follow-up and outcomes

Primary and secondary outcomes were the first diagnosis of respiratory infection and first diagnosis of non-respiratory infection after birth-related hospital discharge, respectively. All infectious disease outcomes were coded according to the International Statistical Classification of Disease and Related Health Problems (ICD-10) 10th revision (Scotland and Finland adopted the ICD-10 in 1996[2]). Data on infections were extracted from Scottish Morbidity Records (SMR01) and from the Finnish Care Register for Health Care (HILMO). The SMR01 includes episode-level data on inpatient and day case records. The HILMO includes episode-level data on in- and outpatient data. Primary and all secondary diagnostic codes were searched for ICD-10 codes of infections. ICD-10 codes were grouped into seven clinical categories in accordance with previous literature (list of ICD-10 codes in the online supplement).[3]

Respiratory infections were defined as either lower respiratory tract infections (LRTI) or upper respiratory tract infections (URTI). LRTIs were defined as microbiologically, clinically, or radiologically diagnosed airway infection in and distal to the bronchi, including influenza with predominantly respiratory features (i.e., pneumonia, bronchiolitis, influenza, whooping cough, bronchitis, and unspecified LRTI). URTIs were defined as infections in the respiratory tract proximal to the vocal cords, external, middle, and inner ear, mastoid, and sinuses (i.e., pharyngitis/tonsillitis/laryngitis, sinusitis, abscesses).

Non-respiratory infection subgroups included invasive bacterial (bacterial isolation from a normally sterile deep site, usually blood, CSF, or bone), gastrointestinal (bacterial, viral, or protozoal gastroenteritis, or clinical diagnosis of infective gastroenteritis as the study period partly pre-dates rapid diagnostics), skin and soft tissue (skin and adnexa, including cellulitis and myositis), genito-urinary (urinary or genital tract, including sexually transmitted diseases), or viral infections (not other specified viral causes). A full list of ICD-10 codes for respiratory and non-respiratory infections used is available in the online supplement and previous publications.[3]

Covariates

Covariates were chosen based on their association with exposure and outcome based on literature review [3-8] and based on a direct acyclic graph (DAG) (Figure E1). Various potential covariates from the DAG could not be included as no data was available for these factors or due to high percentage of missing information. [1] The final model included maternal age at birth (in years, continuous, range 13 – 60 years), parity (continuous, range 0 – 18), maternal smoking at first antenatal appointment/first trimester (current smoker vs previous smoker & never smoked, categorical), maternal diabetes (preexisting diabetes or gestational diabetes vs none, categorical), gestational age at birth (in weeks, continuous, range 28^{+0} – 41^{+6}), country (Scotland or Finland), mode of delivery (planned and unscheduled caesarean section or vaginal including assisted vaginal, categorical), year of birth (discrete, range: Scotland 1997 – 2018, Finland 2006 – 2018), child sex (categorical, female and male) and birthweight centiles (continuous, percentiles adjusted for sex and gestational age [9]).

Statistical Analyses

For all outcomes, ACS-exposed children were compared to ACS non-exposed children. Outcomes for exposure and comparison groups were stratified based on gestational age categories at birth (completed gestation weeks at

© 2025 Decrue F et al. *JAMA Network Open*.

birth) according to WHO definitions.[10] However, moderate-to-late preterm children were divided into two groups, as the clear benefit of ACS treatment until 33^{+6} weeks gestation has been shown,[11,12] whereas the long-term benefit-risk ratio for the children born $34^{+0} – 36^{+6}$ is still unknown. Grouping subsequently included children born very preterm ($28^{+0} – 31^{+6}$ weeks gestation), moderate preterm ($32^{+0} – 33^{+6}$ weeks gestation), late preterm (34^{+0} – 36^{+6} weeks gestation), early-term ($37^{+0} – 38^{+6}$ weeks gestation), full-/late-term ($39^{+0} – 41^{+6}$ weeks gestation). The associations of covariates with ACS exposure and with infectious diseases was assessed with χ^2 tests, t-tests, and univariate analyses of variance.

We calculated the incidence rate of first infectious diseases per 1000 person-years for each gestational group. This was done for each of the infectious disease subgroups (i.e., URTI, LRTI, gastrointestinal, genito-urinary, skin and soft tissue, invasive bacterial, and viral).

Cox (proportional hazard) regression was used to estimate hazard ratio with 95% confidence intervals to hazard of first respiratory infectious disease (any) and first non-respiratory infectious disease (any) in ACS-exposed vs non-exposed offspring, with offspring's age as the time scale. Participants were followed up from discharge from birth-related hospitalisation until first respiratory or non-respiratory infection, or end of follow-up or death, whichever occurred first. We used a simple adjusted model in the first step (adjusted for child sex, year of birth) and in a second step adjusted for all covariates listed above. Cox proportionality assumptions were tested using Kaplan-Meier survival curves. Statistical analyses were conducted using STATA version 16.1 and R 4.2.0.

eResults

Study population and characteristics

1,548,538 (80.3%) mother-child pairs from the Scottish and Finnish registries were included in the study from a total of 1,929,157 (eFigure 2 and eTable 1). Of these, 887,290 (57.3%) were Scottish and 661,248 (42.7%) Finnish participants. In total 49,263 (3.2%) children were exposed to ACS, from which 34,806 (70.7%) were born preterm and 14,457 (29.3%) born term. Anthropometric data and covariates are shown in Table 1 and according to country, term birth and ACS exposure in eTable 2, 3and 4. Median (IQR) age at first episode of infectious disease was 494 days (215 – 1,070 days) for respiratory and 567 days (245 – 1,281 days) for non-respiratory infectious disease, respectively (eTable 5).

Differences between ACS-exposed and non-exposed participants are shown (Table 1, eTable 4). Mothers of ACS-exposed children were older, more often smokers, had higher body mass index (BMI), were more likely to have diabetes (pre-existing or gestational), and more frequently diagnosed with hypertension or preeclampsia than mothers of non-exposed children. ACS-exposed children were born at younger GA, were more often boys, and born via Caesarean section when compared to their non-exposed peers (Table 1).

ICD-10 codes of respiratory infectious diseases

Respiratory infectious diseases include the following International Statistical Classification of Disease and Related Health Problems (ICD) 10th revision (ICD-10) diagnoses:

- **URTI**
  - A36.0 Pharyngeal diphtheria
  - A36.1 Nasopharyngeal diphtheria
  - A36.2 Laryngeal diphtheria
  - B05.3+ Measles complicated by otitis media
  - H65.0 Acute serous otitis media
  - H65.1 Other acute nonsuppurative otitis media
  - H65.2 Chronic serous otitis media
  - H65.3 Chronic mucoid otitis media
  - H65.4 Other chronic nonsuppurative otitis media
  - H65.9 Nonsuppurative otitis media, unspecified
  - H66.0 Acute suppurative otitis media
  - H66.1 Chronic tubotympanic suppurative otitis media
  - H66.2 Chronic atticoantral suppurative otitis media
  - H66.3 Other chronic suppurative otitis media
  - H66.4 Suppurative otitis media, unspecified
  - H66.9 Otitis media, unspecified
  - H67.0 Otitis media in bacterial diseases classified elsewhere
  - H67.8* Otitis media in other diseases classified elsewhere

© 2025 Decrue F et al. *JAMA Network Open*.

- H68.0 Eustachian salpingitis
- H70.0 Acute mastoiditis
- H70.1 Chronic mastoiditis
- H70.2 Petrositis
- H70.8 Other mastoiditis and related conditions
- H70.9 Mastoiditis, unspecified
- H72.0 Central perforation of tympanic membrane
- H72.1 Attic perforation of tympanic membrane
- H72.2 Other marginal perforations of tympanic membrane
- H72.8 Other perforations of tympanic membrane
- H72.9 Perforation of tympanic membrane, unspecified
- H73.0 Acute myringitis
- H75.0 Mastoiditis in infectious and parasitic diseases classified elsewhere
- H83.0 Labyrinthitis
- H92.1 Otorrhoea
- J00 Acute nasopharyngitis [common cold]
- J01.0 Acute maxillary sinusitis
- J01.1 Acute frontal sinusitis
- J01.2 Acute ethmoidal sinusitis
- J01.3 Acute sphenoidal sinusitis
- J01.4 Acute pansinusitis
- J01.8 Other acute sinusitis
- J01.9 Acute sinusitis, unspecified
- J02.0 Streptococcal pharyngitis
- J02.8 Acute pharyngitis due to other specified organisms
- J02.9 Acute pharyngitis, unspecified
- J03.0 Streptococcal tonsillitis
- J03.8 Acute tonsillitis due to other specified organisms
- J03.9 Acute tonsillitis, unspecified
- J04.0 Acute laryngitis
- J04.1 Acute tracheitis
- J04.2 Acute laryngotracheitis
- J05.1 Acute epiglottitis
- J06.0 Acute laryngopharyngitis
- J06.8 Other acute upper respiratory infections of multiple sites
- J06.9 Acute upper respiratory infection, unspecified
- J32.0 Chronic maxillary sinusitis
- J32.1 Chronic frontal sinusitis
- J32.2 Chronic ethmoidal sinusitis
- J32.3 Chronic sphenoidal sinusitis
- J32.4 Chronic pansinusitis
- J32.8 Other chronic sinusitis
- J32.9 Chronic sinusitis, unspecified
- J34.0 Abscess, furuncle and carbuncle of nose
- J35.0 Chronic tonsillitis
- J36 Peritonsillar abscess

© 2025 Decrue F et al. *JAMA Network Open*.

- **LRTI**

- A06.5+ Amebic lung abscess
- A15.0 Tuberculosis of lung
- A15.1 Tuberculosis of lung, confirmed by culture only
- A15.2 Tuberculosis of lung, confirmed histologically
- A15.3 Tuberculosis of lung, confirmed by unspecified means
- A15.4 Tuberculosis of intrathoracic lymph nodes
- A15.5 Tuberculosis of larynx, trachea and bronchus
- A15.6 Tuberculosis pleurisy
- A15.7 Primary respiratory tuberculosis
- A15.8 Other respiratory tuberculosis
- A15.9 Respiratory tuberculosis unspecified, confirmed bacteriologically and histologically
- A16.0 Tuberculosis of lung, bacteriologically and histologically negative
- A16.1 Tuberculosis of lung, bacteriological and histological examination not done
- A16.2 Tuberculosis of lung, without mention of bacteriological or histological confirmation
- A16.3 Tuberculosis of intrathoracic lymph nodes, without mention of bacteriological or histological confirmation
- A16.4 Tuberculosis of larynx, trachea and bronchus, without mention of bacteriological or histological confirmation
- A16.5 Tuberculous pleurisy, without mention of bacteriological or histological confirmation
- A16.7 Primary respiratory tuberculosis, without mention of bacteriological or histological confirmation
- A16.8 Other respiratory tuberculosis, without mention of bacteriological or histological confirmation
- A16.9 Respiratory tuberculosis unspecified, without mention of bacteriological or histological confirmation
- A19.2 Acute miliary tuberculosis, unspecified
- A19.8 Other miliary tuberculosis
- A19.9 Miliary tuberculosis, unspecified
- A20.2 Pneumonic plague
- A21.2 Pulmonary tularemia
- A22.1 Pulmonary anthrax
- A24.0 Glanders
- A31.0 Pulmonary mycobacterial infection
- A37.0 Whooping cough due to Bordetella pertussis
- A37.1 Whooping cough due to Bordetella parapertussis
- A37.8 Whooping cough due to other Bordetella species
- A37.9 Whooping cough, unspecified
- A42.0 Pulmonary actinomycosis
- A48.1 Legionnaires disease
- A70 Chlamydia psittaci infections
- A70+ (no description found)
- B01.2+ Varicella pneumonia
- B05.2+ Measles complicated by pneumonia
- B34.0 Adenovirus infection, unspecified
- B37.1 Pulmonary candidiasis
- B38.0 Acute pulmonary coccidioidomycosis
- B38.1 Chronic pulmonary coccidioidomycosis
- B38.2 Pulmonary coccidioidomycosis, unspecified
- B39.0 Acute pulmonary histoplasmosis capsulati
- B39.1 Chronic pulmonary histoplasmosis capsulati
- B39.2 Pulmonary histoplasmosis capsulati, unspecified
- B39.3 Disseminated histoplasmosis capsulati
- B39.4 Histoplasmosis capsulati, unspecified
- B39.5 Histoplasmosis duboisii
- B39.9 Histoplasmosis, unspecified
- B40.0 Acute pulmonary blastomycosis
- B40.1 Chronic pulmonary blastomycosis
- B40.2 Pulmonary blastomycosis, unspecified
- B40.3 Cutaneous blastomycosis
- B40.7 Disseminated blastomycosis

© 2025 Decrue F et al. *JAMA Network Open*.

- B40.8 Other forms of blastomycosis
- B41.0 Pulmonary paracoccidioidomycosis
- B42.0+ Pulmonary sporotrichosis
- B44.0 Invasive pulmonary aspergillosis
- B44.1 Other pulmonary aspergillosis
- B45.0 Pulmonary cryptococcosis
- B58.3+ Pulmonary toxoplasmosis
- B59 Pneumocystosis
- J05.0 Acute obstructive laryngitis [croup]
- J09 Influenza due to certain identified influenza virus
- J10.0 Influenza with pneumonia, influenza virus identified
- J10.1 Influenza due to other influenza virus with respiratory manifestations
- J11.0 Influenza with pneumonia, virus not identified
- J11.1 Influenza with other respiratory manifestations, virus not identified
- J12.0 Adenoviral pneumonia
- J12.1 Respiratory syncytial virus pneumonia
- J12.2 Parainfluenza virus pneumonia
- J12.3 Human metapneumovirus pneumonia
- J12.8 Other viral pneumonia
- J12.9 Viral pneumonia, unspecified
- J13 Pneumonia due to Streptococcus pneumoniae
- J14 Pneumonia due to Hemophilus influenzae
- J15.0 Pneumonia due to Klebsiella pneumoniae
- J15.1 Pneumonia due to Pseudomonas
- J15.2 Pneumonia due to staphylococcus
- J15.3 Pneumonia due to streptococcus, group B
- J15.4 Pneumonia due to other streptococci
- J15.5 Pneumonia due to Escherichia coli
- J15.6 Pneumonia due to other aerobic Gram-negative bacteria
- J15.7 Pneumonia due to Mycoplasma pneumoniae
- J15.8 Pneumonia due to other specified bacteria
- J15.9 Unspecified bacterial pneumonia
- J16.0 Chlamydial pneumonia
- J16.8 Pneumonia due to other specified infectious organisms
- J17.0* Pneumonia in bacterial diseases classified elsewhere
- J17.1* Pneumonia in viral diseases classified elsewhere
- J17.2* Pneumonia in mycoses
- J17.3 Pneumonia in parasitic diseases
- J17.8* Pneumonia in other diseases classified elsewhere
- J18.0 Bronchopneumonia, unspecified organism
- J18.1 Lobar pneumonia, unspecified
- J18.8 Other pneumonia, unspecified organism
- J18.9 Pneumonia, unspecified
- J20.0 Acute bronchitis due to Mycoplasma pneumoniae
- J20.1 Acute bronchitis due to Hemophilus influenzae
- J20.2 Acute bronchitis due to streptococcus
- J20.3 Acute bronchitis due to coxsackievirus
- J20.4 Acute bronchitis due to parainfluenza virus
- J20.5 Acute bronchitis due to respiratory syncytial virus
- J20.6 Acute bronchitis due to rhinovirus
- J20.7 Acute bronchitis due to echovirus
- J20.8 Acute bronchitis due to other specified organisms
- J20.9 Acute bronchitis, unspecified
- J21.0 Acute bronchiolitis due to respiratory syncytial virus
- J21.1 Acute bronchiolitis due to human megapneumovirus
- J21.8 Acute bronchiolitis due to other specified organisms
- J21.9 Acute bronchiolitis, unspecified
- J22 Unspecified acute lower respiratory infection
- J22.0 (no description found)

© 2025 Decrue F et al. *JAMA Network Open*.

- J40 Bronchitis, not specified as acute or chronic
- J41.0 Simple chronic bronchitis
- J41.1 Mucopurulent chronic bronchitis
- J41.8 Mixed simple and mucopurulent chronic bronchitis
- J42 Unspecified chronic bronchitis
- J44.0 Chronic obstructive pulmonary disease with acute lower respiratory infection
- J47 Bronchiectasis
- J65 Pneumoconiosis associated with tuberculosis
- J85.0 Gangrene and necrosis of lung
- J85.1 Abscess of lung with pneumonia
- J85.2 Abscess of lung without pneumonia
- J86.0 Pyothorax with fistula
- J86.9 Pyothorax without fistula
- P23.0 Congenital pneumonia due to viral agent
- P23.2 Congenital pneumonia due to staphylococcus
- P23.3 Congenital pneumonia due to staphylococcus, group B
- P23.4 Congenital pneumonia due to Escherichia coli
- P23.5 Congenital pneumonia due to Pseudomonas
- P23.6 Congenital pneumonia due to other bacterial agents
- P23.8 Congenital pneumonia due to other organisms
- P23.9 Congenital pneumonia, unspecified

© 2025 Decrue F et al. *JAMA Network Open*.

ICD-10 list of non-respiratory infectious diseases

- **Bacterial invasive**

- A02.1 Salmonella sepsis
- A17.0+ Tuberculous meningitis
- A17.1+ Meningeal tuberculoma
- A17.8+ Other tuberculosis of nervous system
- A17.9+ Tuberculosis of nervous system, unspecified
- A18.0+ Tuberculosis of bones and joints
- A19.0 Acute miliary tuberculosis of a single specified site
- A19.1 Acute miliary tuberculosis of multiple sites
- A20.3 Plague meningitis
- A20.7 Septicemic plague
- A21.7 Generalized tularaemia
- A22.7 Anthrax sepsis
- A23.0 Brucellosis due to Brucella melitensis
- A23.1 Brucellosis due to Brucella abortus
- A23.2 Brucellosis due to Brucella suis
- A23.3 Brucellosis due to Brucella canis
- A23.8 Other brucellosis
- A23.9 Brucellosis, unspecified
- A24.1 Acute and fulminating melioidosis
- A25.0 Spirillosis
- A25.1 Streptobacillosis
- A25.9 Rat-bite fever, unspecified
- A32.1 Listerial meningitis and meningoencephalitis
- A32.7 Listerial sepsis
- A39.0+ Meningococcal meningitis
- A39.1+ Waterhouse-Friderichsen syndrome
- A39.2 Acute meningococcaemia
- A39.3 Chronic meningococcaemia
- A39.4 Meningococcemia, unspecified
- A39.5 Meningococcal heart disease
- A39.8 Other meningococcal infections
- A39.8+Other meningococcal infections
- A39.9 Meningococcal infection, unspecified
- A40.0 Sepsis due to streptococcus, group A
- A40.1 Sepsis due to streptococcus, group B
- A40.2 Sepsis due to streptococcus, group D
- A40.3 Sepsis due to Streptococcus pneumoniae
- A40.8 Other streptococcal sepsis
- A40.9 Streptococcal sepsis, unspecified
- A41.0 Sepsis due to Staphylococcus aureus
- A41.1 Sepsis due to other specified staphylococcus
- A41.2 Sepsis due to unspecified staphylococcus
- A41.3 Sepsis due to Hemophilus influenzae
- A41.4 Sepsis due to anaerobes
- A41.51 Sepsis due to Escherichia coli [E. Coli]
- A41.52 Sepsis due to Pseudomonas
- A41.58 Sepsis due to other Gram-negative organisms
- A41.8 Other specified septicaemia
- A41.9 Sepsis, unspecified
- A44.0 Systemic bartonellosis
- A48.3 Toxic shock syndrome
- A52.0+ Cardiovascular syphilis (I98.0*)
- A52.1 Symptomatic neurosyphilis
- A52.1+ (no description found)
- A52.2 Asymptomatic neurosyphilis
- A52.3 Neurosyphilis, unspecified
- A52.7 Other symptomatic late syphilis

© 2025 Decrue F et al. *JAMA Network Open*.

- A52.7+ (no description found)
- A52.8 Late syphilis, latent
- A65 Nonvenereal syphilis
- A74.0 Chlamydial conjunctivitis
- A74.8 Other chlamydial diseases
- A78 Q fever
- A79.0 Trench fever
- A79.1 Rickettsialpox due to Rickettsia akari
- A79.8 Other specified rickettsioses
- A79.9 Rickettsiosis, unspecified
- B95.1 Streptococcus, group B, as the cause of diseases classified elsewhere
- G00.0 Haemophilus meningitis
- G00.1 Pneumococcal meningitis
- G00.1* (no description found)
- G00.2 Streptococcal meningitis
- G00.3 Staphylococcal meningitis
- G00.8 Other bacterial meningitis
- G00.9 Bacterial meningitis, unspecified
- G01 Meningitis in bacterial diseases classified elsewhere
- G03.1 Chronic meningitis
- G05.0 Encephalitis, myelitis and encephalomyelitis in bacterial diseases classified elsewhere
- G06.0 Intracranial abscess and granuloma
- G06.1 Intraspinal abscess and granuloma
- G06.2 Extradural and subdural abscess, unspecified
- G07 Intracranial and intraspinal abscess and granuloma in disease classified elsewhere
- I30.1 Infective pericarditis
- I32.0 Pericarditis in bacterial diseases classified elsewhere
- I33.0 Acute and subacute infective endocarditis
- I41.0 Myocarditis in bacterial diseases classified elsewhere
- J39.0 Retropharyngeal and parapharyngeal abscess
- J39.1 Other abscess of pharynx
- J85.3 Abscess of mediastinum
- M00.90 Pyogenic arthritis, unspecified, multiple sites
- M00.91 Pyogenic arthritis, unspecified, shoulder region
- M00.92 Pyogenic arthritis, unspecified, upper arm
- M00.93 Pyogenic arthritis, unspecified, forearm
- M00.94 Pyogenic arthritis, unspecified, hand
- M00.95 Pyogenic arthritis, unspecified, pelvic region and thigh
- M00.96 Pyogenic arthritis, unspecified, lower leg
- M00.97 Pyogenic arthritis, unspecified, ankle and foot
- M00.98 Pyogenic arthritis, unspecified, other site
- M00.99 Pyogenic arthritis, unspecified, site unspecified
- M01.0 Meningococcal arthritis
- M01.1 Tuberculous arthritis
- M46.2 Osteomyelitis of vertebra
- M46.3 Infection of intervertebral disc (pyogenic)
- M46.4 Discitis, unspecified
- M46.5 Other infective spondylopathies
- M49.0 Tuberculosis of spine
- M49.1 Brucella spondylitis
- M49.2 Enterobacterial spondylitis
- M49.3 Spondylopathy in other infectious and parasitic diseases classified elsewhere
- M72.6 Necrotizing fasciitis
- M73.0 Gonococcal bursitis
- M73.1 Syphilitic bursitis
- M86.0 Acute haematogenous osteomyelitis
- M86.10 Other acute osteomyelitis, unspecified site
- M86.11 Other acute osteomyelitis, shoulder region
- M86.12 Other acute osteomyelitis, upper arm

- M86.13 Other acute osteomyelitis, forearm
- M86.14 Other acute osteomyelitis, hand
- M86.15 Other acute osteomyelitis, pelvic region and thigh
- M86.16 Other acute osteomyelitis, lower leg
- M86.17 Other acute osteomyelitis, ankle and foot
- M86.18 Other acute osteomyelitis, other site
- M86.19 Other acute osteomyelitis, multiple sites
- M86.2 Subacute osteomyelitis
- M86.4 Chronic osteomyelitis with draining sinus
- M86.5 Other chronic osteomyelitis
- M86.60 Other chronic osteomyelitis, unspecified site
- M86.67 Other chronic osteomyelitis, ankle and foot
- M86.68 Other chronic osteomyelitis, other site
- M86.69 Other chronic osteomyelitis, multiple sites
- M86.8 Other osteomyelitis
- M86.90 Unspecified osteomyelitis, multiple sites
- M86.91 Unspecified osteomyelitis, shoulder region
- M86.92 Unspecified osteomyelitis, upper arm
- M86.93 Unspecified osteomyelitis, forearm
- M86.94 Unspecified osteomyelitis, hand
- M86.95 Unspecified osteomyelitis, pelvic region and thigh
- M86.96 Unspecified osteomyelitis, lower leg
- M86.97 Unspecified osteomyelitis, ankle and foot
- M86.98 Unspecified osteomyelitis, other site
- M86.99 Unspecified osteomyelitis, site unspecified
- M90.0 Tuberculosis of bone
- P36.0 Sepsis of newborn due to streptococcus, group B
- P36.1 Sepsis of newborn due to other and unspecified streptococci
- P36.2 Sepsis of newborn due to Staphylococcus aureus
- P36.3 Sepsis of newborn due to other and unspecified staphylococci
- P36.4 Sepsis of newborn due to Escherichia coli
- P36.5 Sepsis of newborn due to anaerobes
- P36.8 Other bacterial sepsis of newborn
- P36.9 Bacterial sepsis of newborn, unspecified
- P37.0 Congenital tuberculosis
- P37.2 Neonatal (disseminated) listeriosis

© 2025 Decrue F et al. *JAMA Network Open*.

- **Gastrointestinal infections**

- A00.0 Cholera due to Vibrio cholerae 01, biovar cholerae
- A00.1 Cholera due to Vibrio cholerae 01, biovar eltor
- A00.9 Cholera, unspecified
- A01.0 Typhoid fever
- A01.1 Paratyphoid fever A
- A01.2 Paratyphoid fever B
- A01.3 Paratyphoid fever C
- A01.4 Paratyphoid fever, unspecified
- A02.0 Salmonella enteritis
- A02.2+ Localised salmonella infections
- A02.8 Other specified salmonella infections
- A02.9 Salmonella infection, unspecified
- A03.0 Shigellosis due to Shigella dysenteriae
- A03.1 Shigellosis due to Shigella flexneri
- A03.2 Shigellosis due to Shigella boydii
- A03.3 Shigellosis due to Shigella sonnei
- A03.8 Other shigellosis
- A03.9 Shigellosis, unspecified
- A04.0 Enteropathogenic Escherichia coli infection
- A04.1 Enterotoxigenic Escherichia coli infection
- A04.2 Enteroinvasive Escherichia coli infection
- A04.3 Enterohemorrhagic Escherichia coli infection
- A04.4 Other intestinal Escherichia coli infections
- A04.5 Campylobacter enteritis
- A04.6 Enteritis due to Yersinia enterocolitica
- A04.7 Enterocolitis due to Clostridium difficile
- A04.8 Other specified bacterial intestinal infections
- A04.9 Bacterial intestinal infection, unspecified
- A05.0 Foodborne staphylococcal intoxication
- A05.1 Botulism food poisoning
- A05.2 Foodborne Clostridium perfringens [Clostridium welchii] intoxication
- A05.3 Foodborne Vibrio parahaemolyticus intoxication
- A05.4 Foodborne Bacillus cereus intoxication
- A05.8 Other specified bacterial foodborne intoxications
- A05.9 Bacterial foodborne intoxication, unspecified
- A06.0 Acute amebic dysentery
- A06.1 Chronic intestinal amebiasis
- A06.2 Amebic nondysenteric colitis
- A06.3 Amoeboma of intestine
- A06.4 Amebic liver abscess
- A06.9 Amebiasis, unspecified
- A07.0 Balantidiasis
- A07.1 Giardiasis [lambliasis]
- A07.2 Cryptosporidiosis
- A07.3 Isosporiasis
- A07.8 Other specified protozoal intestinal diseases
- A07.9 Protozoal intestinal disease, unspecified
- A08.0 Rotaviral enteritis
- A08.1 Acute gastroenteropathy due to Norwalk agent
- A08.2 Adenoviral enteritis
- A08.3 Other viral enteritis
- A08.4 Viral intestinal infection, unspecified
- A08.5 Other specified intestinal infections
- A09 Infectious gastroenteritis and colitis, unspecified
- A09.0 Other and unspecified gastroenteritis and colitis of infectious origin
- A09.9 Gastroenteritis and colitis of unspecified origin
- A21.3 Gastrointestinal tularemia
- A22.2 Gastrointestinal anthrax

- B37.88 Candidiasis of other sites
- B71.0 Hymenolepiasis
- B71.9 Cestode infection, unspecified
- B76.0 Ancylostomiasis
- B78.9 Strongyloidiasis, unspecified
- B81.0 Anisakiasis
- B96.81 Helicobacter pylori [H. pylori] as the cause of diseases classified to other chapters
- K52.8 Other specified noninfective gastroenteritis and colitis

© 2025 Decrue F et al. *JAMA Network Open*.

Skin and soft tissue

- A31.1 Cutaneous mycobacterial infection
- A46 Erysipelas
- B00.0 Eczema herpeticum
- B35.0 Tinea barbae and tinea capitis
- B35.1 Tinea unguium
- B35.2 Tinea manuum
- B35.3 Tinea pedis
- B35.4 Tinea corporis
- B35.5 Tinea imbricate
- B35.6 Tinea cruris
- B35.8 Other dermatophytoses
- B35.9 Dermatophytosis, unspecified
- B36.0 Pityriasis versicolour
- B36.1 Tinea nigra
- B36.2 White piedra
- B36.3 Black piedra
- B36.8 Other specified superficial mycoses
- B37.2 Candidiasis of skin and nail
- B85.0 Pediculosis due to Pediculus humanus capitis
- B85.1 Pediculosis due to Pediculus humanus corporis
- B85.2 Pediculosis, unspecified
- B85.3 Phthiriasis
- B85.4 Mixed pediculosis and phthiriasis
- B86 Scabies
- B87.9 Myiasis, unspecified
- B88.0 Other acariasis
- B88.1 Tungiasis [sandflea infestation]
- B88.2 Other arthropod infestations
- B88.3 External hirudiniasis
- B88.8 Other specified infestations
- B88.9 Infestation, unspecified
- H60.3 Other infective otitis externa
- L00 Staphylococcal scalded skin syndrome
- L01.0 Impetigo [any organism] [any site]
- L02.0 Cutaneous abscess, furuncle and carbuncle of face
- L02.1 Cutaneous abscess, furuncle and carbuncle of neck
- L02.2 Cutaneous abscess, furuncle and carbuncle of trunk
- L02.3 Cutaneous abscess, furuncle and carbuncle of buttock
- L02.4 Cutaneous abscess, furuncle and carbuncle of limb
- L02.8 Cutaneous abscess, furuncle and carbuncle of other sites
- L02.9 Cutaneous abscess, furuncle and carbuncle, unspecified
- L03.01 Cellulitis of finger
- L03.02 Cellulitis of toe
- L03.10 Cellulitis of upper limb
- L03.11 Cellulitis of lower limb
- L03.2 Cellulitis of face
- L03.3 Cellulitis of trunk
- L03.8 Cellulitis of other sites
- L03.9 Cellulitis, unspecified
- L05.0 Pilonidal cyst with abscess
- L05.9 Pilonidal cyst without abscess
- L08.0 Pyoderma
- L08.1 Erythrasma
- L08.8 Other specified local infections of skin and subcutaneous tissue
- L08.9 Local infection of the skin and subcutaneous tissue, unspecified
- L13.0 Dermatitis herpetiformis
- L30.3 Infective dermatitis
- L88 Pyoderma gangrenosum

© 2025 Decrue F et al. *JAMA Network Open*.

- M60.09 Infective myositis, multiple sites
- M63.0 Myositis in bacterial diseases classified elsewhere
- M63.1 Myositis in protozoal and parasitic infections classified elsewhere
- M63.2 Myositis in other infectious diseases classified elsewhere
- M65.0 Abscess of tendon sheath
- M65.1 Other infective (teno)synovitis
- M68.0 Synovitis and tenosynovitis in bacterial diseases classified elsewhere
- M71.0 Abscess of bursa
- M71.1 Other infective bursitis
- O91.00 Infection of nipple associated with childbirth, without mention of attachment difficulty
- O91.10 Abscess of breast associated with childbirth, without mention of attachment difficulty
- P39.0 Neonatal infective mastitis
- P39.4 Neonatal skin infection

© 2025 Decrue F et al. *JAMA Network Open*.

- **Genito-urinary infections**

- A36.8+ (no description found)
- A51.0 Primary genital syphilis
- A51.1 Primary anal syphilis
- A51.2 Primary syphilis of other sites
- A51.3 Secondary syphilis of skin and mucous membranes
- A51.3+ (no description found)
- A51.4 Other secondary syphilis
- A51.5 Early syphilis, latent
- A51.9 Early syphilis, unspecified
- A52.9 Late syphilis, unspecified
- A53.0 Latent syphilis, unspecified as early or late
- A53.9 Syphilis, unspecified
- A54.0 Gonococcal infection of lower genito-urinary tract without periurethral or accessory gland abscess
- A54.1 Gonococcal infection of lower genito-urinary tract with periurethral and accessory gland abscess
- A54.2+ Gonococcal pelviperitonitis and other gonococcal genito-urinary infections
- A54.3 Gonococcal infection of eye
- A54.3+ (no description found)
- A54.4+ Gonococcal infection of musculoskeletal system
- A54.5 Gonococcal pharyngitis
- A54.6 Gonococcal infection of anus and rectum
- A54.8 Other gonococcal infections
- A54.8+ (no description found)
- A54.9 Gonococcal infection, unspecified
- A55 Chlamydial lymphogranuloma (venereum)
- A56.0 Chlamydial infection of lower genito-urinary tract
- A56.1 Chlamydial infection of pelviperitoneum and other genito-urinary organs
- A56.2 Chlamydial infection of genito-urinary tract, unspecified
- A56.3 Chlamydial infection of anus and rectum
- A56.4 Chlamydial infection of pharynx
- A56.8 Sexually transmitted chlamydial infection of other sites
- A57 Chancroid
- A58 Granuloma inguinale
- A59.0 Urogenital trichomoniasis
- A59.0+ (no description found)
- A60.0 Herpesviral infection of genitalia and urogenital tract
- A60.1 Herpesviral infection of perianal skin and rectum
- A60.9 Anogenital herpesviral infection, unpsecified
- A63.0 Anogenital (venereal) warts
- A63.8 Other specified predominantly sexually transmitted diseases
- A64 Unspecified sexually transmitted disease
- B37.3+ Candidiasis of vulva and vagina
- B37.4+ Candidiasis of other urogenital sites
- N13.6 Pyonephrosis
- N15.1 Renal and perinephric abscess
- N30.0 Acute cystitis
- N30.8 Other cystitis, abscess of bladder
- N34.0 Urethral abscess
- N35.1 Postinfective urethral stricture, not elsewhere classified
- N39.0 Urinary tract infection, site not specified
- N41.0 Acute prostatitis
- N41.2 Abscess of prostate
- N43.1 Infected hydrocele
- N45.0 Orchitis, epididymitis and epididymo-orchitis with abscess
- N45.9 Orchitis, epididymitis and epididymo-orchits without abscess
- N51.2 Balanitis in diseases classified elsewhere
- N70.0 Acute salpingitis and oophoritis
- N73.0 Acute parametritis and pelvic cellulitis
- N73.1 Chronic parametritis and pelvic cellulitis

© 2025 Decrue F et al. *JAMA Network Open*.

- N73.2 Unspecified parametritis and pelvic cellulitis
- N73.3 Female acute pelvic peritonitis
- N73.4 Female chronic pelvic peritonitis
- N73.9 Female pelvic inflammatory disease, unspecified
- N74.2 Female syphilitic pelvic inflammatory disease
- N74.3 Female gonococcal pelvic inflammatory disease
- N74.4 Female chlamydial pelvic inflammatory disease
- N75.0 Cyst of Bartholins gland
- N75.1 Abscess of Bartholins gland
- N76.0 Acute vaginitis
- N76.4 Abscess of vulva
- O03.0 Spontaneous abortion, incomplete, complicated by genital tract and pelvic infection
- O03.5 Spontaneous abortion, complete or unspecified, complicated by genital tract and pelvic infection
- O08.0 Genital tract and pelvic infection following ectopic and molar pregnancy
- O26.4 Herpes gestationis
- O86.2 Urinary tract infection following delivery
- O98.1 Syphilis complicating pregnancy, childbirth and the puerperium
- O98.2 Gonorrhea complicating pregnancy, childbirth and the puerperium
- P39.3 Neonatal urinary tract infection

© 2025 Decrue F et al. *JAMA Network Open*.

- **Viral infections**

- A80.1 Acute paralytic poliomyelitis, wild virus, imported
- A80.2 Acute paralytic poliomyelitis, wild virus, indigenous
- A80.3 Acute paralytic poliomyelitis, other and unspecified
- A80.4 Acute nonparalytic poliomyelitis
- A80.9 Acute poliomyelitis, unspecified
- A81.8 Other atypical virus infections of central nervous system
- A81.9 Atypical virus infection of central nervous system, unspecified
- A82.0 Sylvatic rabies
- A82.1 Urban rabies
- A82.9 Rabies, unspecified
- A83.0 Japanese encephalitis
- A83.1 Western equine encephalitis
- A83.2 Eastern equine encephalitis
- A83.3 St Louis encephalitis
- A83.4 Australian encephalitis
- A83.5 California encephalitis
- A83.6 Rocio virus disease
- A83.8 Other mosquito-borne viral encephalitis
- A83.9 Mosquito-borne viral encephalitis, unspecified
- A84.0 Far Eastern tick-borne encephalitis [Russian spring-summer encephalitis]
- A84.1 Central European tick-borne encephalitis
- A84.8 Other tick-borne viral encephalitis
- A84.9 Tick-borne viral encephalitis, unspecified
- A85.0 Enteroviral encephalitis
- A85.1 Adenoviral encephalitis
- A85.2 Arthropod -borne viral encephalitis, unspecified
- A85.8 Other specified viral encephalitis
- A86 Unspecified viral encephalitis
- A87.0+ Enteroviral meningitis
- A87.1+ Adenoviral meningitis
- A87.2 Lymphocytic choriomeningitis
- A87.8 Other viral meningitis
- A87.9 Viral meningitis, unspecified
- A88.0 Enteroviral exanthematous fever
- A88.8 Other specified viral infections of central nervous system
- A89 Unspecified viral infection of central nervous system
- A90 Dengue fever [classical dengue]
- A91 Dengue haemorrhagic fever
- A92.0 Chikungunya virus disease
- A92.1 O'nyong - nyong fever
- A92.2 Venezuelan equine fever
- A92.3 West Nile virus infection
- A92.4 Rift Valley fever
- A92.8 Other specified mosquito -borne viral fevers
- A92.9 Mosquito -borne viral fever, unspecified
- A93.0 Oropouche virus disease
- A93.2 Colorado tick fever
- A93.8 Other specified arthropod -borne viral fevers
- A94 Unspecified arthropod -borne viral fever
- A95.0 Sylvatic yellow fever
- A95.1 Urban yellow fever
- A95.9 Yellow fever, unspecified
- A96.0 Junin haemorrhagic fever
- A96.1 Machupo haemorrhagic fever
- A96.2 Lassa fever
- A96.8 Other arenaviral hemorrhagic fevers
- A96.9 Arenaviral haemorrhagic fever, unspecified
- A98.0 Crimean -Congo hemorrhagic fever

- A98.1 Omsk hemorrhagic fever
- A98.2 Kyasanur Forest disease
- A98.3 Marburg virus disease
- A98.4 Ebola virus disease
- A98.5 Hemorrhagic fever with renal syndrome
- A98.8 Other specified viral haemorrhagic fevers
- A99 Unspecified viral haemorrhagic fever
- B00.1 Herpesviral vesicular dermatitis
- B00.2 Herpesviral gingivostomatitis and pharyngotonsillitis
- B00.3+ Herpesviral meningitis
- B00.4+ Herpesviral encephalitis
- B00.5+ Herpesviral ocular disease
- B00.7 Disseminated herpesviral disease
- B00.8 Other forms of herpesviral infection
- B00.9 Herpesviral infection, unspecified
- B01.0 Varicella meningitis
- B01.1+ Varicella encephalitis (G05.1*)
- B01.8 Varicella with other complications
- B01.9 Varicella without complication
- B02.0 Zoster encephalitis
- B02.1+ Zoster meningitis
- B02.2+ Zoster with other nervous system involvement
- B02.3+ Zoster ocular disease
- B02.7 Disseminated zoster
- B02.8 Zoster with other complications
- B02.9 Zoster without complications
- B03 Smallpox
- B04 Monkeypox
- B05.0+ Measles complicated by encephalitis
- B05.1 Measles complicated by meningitis
- B05.4 Measles with intestinal complications
- B05.8 Measles with other complications
- B05.9 Measles without complication
- B06.0+ Rubella with neurological complications
- B06.8 Rubella with other complications
- B06.9 Rubella without complication
- B07 Viral warts
- B08.0 Other orthopoxvirus infections
- B08.1 Molluscum contagiosum
- B08.2 Exanthema subitum [sixth disease]
- B08.3 Erythema infectiosum [fifth disease]
- B08.4 Enteroviral vesicular stomatitis with exanthem
- B08.5 Enteroviral vesicular pharyngitis
- B08.8 Other specified viral infections characterized by skin and mucous membrane lesions
- B09 Unspecified viral infection characterized by skin and mucous membrane lesions
- B15.0 Hepatitis A with hepatic coma
- B15.9 Hepatitis A without hepatic coma
- B16.0 Acute hepatitis B with delta-agent with hepatic coma
- B16.1 Acute hepatitis B with delta-agent without hepatic coma
- B16.2 Acute hepatitis B without delta-agent with hepatic coma
- B16.9 Acute hepatitis B without delta-agent and without hepatic coma
- B17.0 Acute delta-(super) infection of hepatitis B carrier
- B17.1 Acute hepatitis C
- B17.2 Acute hepatitis E
- B17.8 Other specified acute viral hepatitis
- B17.9 Acute viral hepatitis, unspecified
- B18.0 Chronic viral hepatitis B with delta-agent
- B18.1 Chronic viral hepatitis B without delta-agent
- B18.2 Chronic viral hepatitis C

© 2025 Decrue F et al. *JAMA Network Open*.

- B18.8 Other chronic viral hepatitis
- B18.9 Chronic viral hepatitis, unspecified
- B19.0 Unspecified viral hepatitis with hepatic coma
- B19.9 Unspecified viral hepatitis without hepatic coma
- B20.0 HIV disease resulting in mycobacterial infection
- B20.1 HIV disease resulting in other bacterial infections
- B20.2 HIV disease resulting in cytomegaloviral disease
- B20.3 HIV disease resulting in other viral infections
- B20.4 HIV disease resulting in candidiasis
- B20.5 HIV disease resulting in other mycoses
- B20.6 HIV disease resulting in Pneumocystis jirovecii pneumonia
- B20.7 HIV disease resulting in multiple infections
- B20.8 HIV disease resulting in other infectious and parasitic diseases
- B20.9 HIV disease resulting in unspecified infectious or parasitic
- B21.0 HIV disease resulting in Kaposi sarcoma
- B21.1 HIV disease resulting in Burkitt lymphoma
- B21.2 HIV disease resulting in other types of non-Hodgkin lymphoma
- B21.3 HIV disease resulting in other malignant neoplasms of lymphoid, haematopoietic and related tissue
- B21.7 HIV disease resulting in multiple malignant neoplasms
- B21.8 HIV disease resulting in other malignant neoplasms
- B21.9 HIV disease resulting in unspecified malignant neoplasm
- B22.0 HIV disease resulting in encephalopathy
- B22.1 HIV disease resulting in lymphoid interstitial pneumonitis
- B22.2 HIV disease resulting in wasting syndrome
- B22.7 HIV disease resulting in multiple diseases classified elsewhere
- B23.0 Acute HIV infection syndrome
- B23.1 HIV disease resulting in (persistent) generalized lymphadenopathy
- B23.2 HIV disease resulting in haematological and immunological abnormalities, not elsewhere classified
- B23.8 HIV disease resulting in other specified conditions
- B24 Unspecified human immunodeficiency virus [HIV] disease
- B25.0 Cytomegaloviral pneumonitis
- B25.1 Cytomegaloviral hepatitis
- B25.2 Cytomegaloviral pancreatitis
- B25.8 Other cytomegaloviral diseases
- B25.9 Cytomegaloviral disease, unspecified
- B26.0+ Mumps orchitis
- B26.1+ Mumps meningitis
- B26.2+ Mumps encephalitis
- B26.3+ Mumps pancreatitis
- B26.8 Mumps with other complications
- B26.8+ (no description found)
- B26.9 Mumps without complication
- B27.0 Gammaherpesviral mononucleosis
- B27.1 Cytomegaloviral mononucleosis
- B27.8 Other infectious mononucleosis
- B27.9 Infectious mononucleosis, unspecified
- B30.0+ Keratoconjunctivitis due to adenovirus
- B30.1+ Conjunctivitis due to adenovirus
- B30.2+ (no description found)
- B30.3+ Acute epidemic hemorrhagic conjunctivitis (enteroviral)
- B30.8+ Other viral conjunctivitis
- B30.9 Viral conjunctivitis, unspecified
- B33.0 Epidemic myalgia
- B33.1 Ross River disease
- B33.2 Viral carditis
- B33.3 Retrovirus infections, not elsewhere classified
- B33.4 Hantavirus (cardio-) pulmonary syndrome

© 2025 Decrue F et al. *JAMA Network Open*.

- B33.8 Other specified viral diseases
- B34.1 Enterovirus infection, unspecified
- B34.2 Coronavirus infection, unspecified site
- B34.3 Parvovirus infection, unspecified site
- B34.4 Papovavirus infection, unspecified
- B34.8 Other viral infections of unspecified site
- B34.9 Viral infection, unspecified
- B97.0 Adenovirus as the cause of diseases classified to other chapters
- B97.1 Enterovirus as the cause of diseases classified to other chapters
- B97.2 Coronavirus as the cause of diseases classified to other chapters
- B97.3 Retrovirus as the cause of diseases classified to other chapters
- B97.4 Respiratory syncytial virus as the cause of diseases classified to other chapters
- B97.5 Retrovirus as the cause of diseases classified to other chapters
- B97.6 Parvovirus as the cause of diseases classified to other chapters
- B97.7 Papillomavirus as the cause of diseases classified to other chapters
- B97.8 Other viral agents as the cause of diseases classified to other chapters
- G02.0* Meningitis in viral diseases classified elsewhere
- G05.1* Encephalitis, myelitis and encephalomyelitis in viral diseases classified elsewhere
- H19.1 Herpesviral keratitis and keratoconjunctivitis
- I41.1 Myocarditis in viral diseases classified elsewhere
- J10.8 Influenza with other manifestations, influenza virus identified
- J11 Influenza, virus not identified
- J11.8 Influenza with other manifestations, virus not identified
- M01.4 Rubella arthritis
- M01.50* Arthritis in other viral diseases classified elsewhere, multiple sites
- M01.51* Arthritis in other viral diseases classified elsewhere, shoulder region
- M01.52* Arthritis in other viral diseases classified elsewhere, upper arm
- M01.53* Arthritis in other viral diseases classified elsewhere, forearm
- M01.54* Arthritis in other viral diseases classified elsewhere, hand
- M01.55* Arthritis in other viral diseases classified elsewhere, pelvic region and thigh
- M01.56* Arthritis in other viral diseases classified elsewhere, lower leg
- M01.57* Arthritis in other viral diseases classified elsewhere, ankle and foot
- M01.58* Arthritis in other viral diseases classified elsewhere, other site
- M01.59* Arthritis in other viral diseases classified elsewhere, site unspecified
- O98.4 Viral hepatitis complicating pregnancy, childbirth and the puerperium
- O98.5 Other viral diseases complicating pregnancy, childbirth and the puerperium
- P35.0 Congenital rubella syndrome
- P35.1 Congenital cytomegalovirus infection
- P35.2 Congenital herpesviral [herpes simples] infection
- P35.3 Congenital viral hepatitis
- P35.8 Other congenital viral diseases
- P35.9 Congenital viral disease, unspecified
- Z21 Asymptomatic human immunodeficiency virus [HIV] infection status

References

1. Frier EM, Lin C, Reynolds RM, et al. Consortium for the Study of Pregnancy Treatments (Co-OPT): An international birth cohort to study the effects of antenatal corticosteroids. *PloS one* 2023; **18**(3): e0282477.
2. Tomasson K. Implementation of ICD-10 in the Nordic countries. *Nordic Journal of Psychiatry* 2009; **53**: 5-9.
3. Miller JE, Hammond GC, Strunk T, et al. Association of gestational age and growth measures at birth with infection-related admissions to hospital throughout childhood: a population-based, data-linkage study from Western Australia. *The Lancet Infectious Diseases* 2016; **16**(8): 952-61.
4. Moore HC, de Klerk N, Richmond P, Lehmann D. A retrospective population-based cohort study identifying target areas for prevention of acute lower respiratory infections in children. *BMC public health* 2010; **10**(1): 757.
5. Melamed N, Shah J, Soraisham A, et al. Association Between Antenatal Corticosteroid Administration-to-Birth Interval and Outcomes of Preterm Neonates. *Obstet Gynecol* 2015; **125**(6): 1377-84.
6. Broughton S, Roberts A, Fox G, et al. Prospective study of healthcare utilisation and respiratory morbidity due to RSV infection in prematurely born infants. *Thorax* 2005; **60**(12): 1039-44.
7. Videholm S, Kostenniemi U, Lind T, Silfverdal S-A. Perinatal factors and hospitalisations for severe childhood infections: a population-based cohort study in Sweden. *BMJ Open* 2021; **11**(10): e054083.
8. Räikkönen K, Gissler M, Kajantie E. Associations Between Maternal Antenatal Corticosteroid Treatment and Mental and Behavioral Disorders in Children. *Jama* 2020; **323**(19): 1924-33.
9. Norris T, Seaton SE, Manktelow BN, et al. Updated birth weight centiles for England and Wales. *Arch Dis Child Fetal Neonatal Ed* 2018; **103**(6): F577-f82.
10. World Health Organization (WHO). Preterm birth. 2018. http://www.who.int/news-room/fact-sheets/detail/preterm-birth (accessed 12.07.2018.
11. American College of Obstetricians and Gynecologists' Committee (ACOG). Committee Opinion No. 713: Antenatal Corticosteroid Therapy for Fetal Maturation. *Obstet Gynecol* 2017; **130**(2): e102-e9.
12. McGoldrick E, Stewart F, Parker R, Dalziel SR. Antenatal corticosteroids for accelerating fetal lung maturation for women at risk of preterm birth. *Cochrane Database of Systematic Reviews* 2020; (12).

© 2025 Decrue F et al. *JAMA Network Open*.

eTable 1. Descriptive characteristics according to country.

	Combined			Finland			Scotland		
	**n/mean**	**%/SD**	**missing information**	**n/mean**	**%/SD**	**missing information**	**n/mean**	**%/SD**	**missing information**
**Total**	1,548,538	NA	NA	661,248	NA	NA	887,290	NA	NA
**Antenatal corticosteroids**	49,263	3·18	0	17,240	2·61	0	32,023	3·61	0
**Preterm (28-36w GA)**	86,482	5·58	0	32,965	4·99	0	53,517	6·03	0
**Term (37-41w GA)**	1,462,056	94·42	0	628,283	95·01	0	833,773	93·97	0
**Maternal age, y**	29·4	5·70	<5	29·9	5·30	0	29·1	6·00	<5
**Maternal BMI**	25·2	5·30	299,061	24·5	4·90	14,898	26·1	5·70	284,163
**Parity**			5,761			105			5,656
**Parity 0**	653,672	42·20	NA	268,272	40·60	NA	385,400	43·40	NA
**Parity 1**	537,323	34·70	NA	226,995	34·30	NA	310,328	35·00	NA
**Parity 2**	221,177	14·30	NA	98,482	14·90	NA	122,695	13·80	NA
**Parity ≥3**	124,844	8·10	NA	67,394	10·20	NA	63,211	7·10	NA
**Smokers**	278,762	18·00	56,678	95,473	14·44	19,326	183,289	20·66	37,352
**Any diabetes**	98,177	6·34	105,920	78,093	11·81	0	20,084	2·26	105,920
**GDM**	83,513	5·39	858,065	73,706	11·15	0	9,807	1·11	858,065
**Hypertension**	8,499	0·55	864,421	5,735	0·87	0	2,764	0·31	864,421
**Preeclampsia**	62,275	4·02	838,511	30,932	4·68	0	31,343	3·53	838,511
**Caesarean section**	338,741	21·87	<5	104,732	15·84	<5	234,009	26·37	<5
**Birthweight, g**	3,443·9	551·4	1,442	3,502·4	529·0	215	3,400·2	563·6	1,227
**Female**	759,082	49·02	95	326,121	49·32	0	432,961	48·80	95
**APGAR <7**	24,490	1·58	92,862	12,474	1·89	80,243	12,016	1·35	12,619
**NICU admission**	143,633	9·28	14,916	69,488	10·51	0	74,145	8·36	14,916
**Gestational age at birth, w**	39·2	1·70	0	39·3	1·60	0	39·2	1·70	0

Abbreviations: BMI = body mass index, g = grams, GDM = gestational diabetes mellitus, n = number, NICU admission = neonatal intensive care unit admission, SD = standard deviation, w = weeks, w GA = weeks gestational age, y = years

© 2025 Decrue F et al. *JAMA Network Open*.

eTable 2. Number of excluded children.

	Combined		Finland		Scotland		Preterm		Term	
	**n**	**%**	**n**	**%**	**n**	**%**	**n**	**%**	**n**	**%**
**Total (before exclusion)**	1,929,157	100	744,536	38·6	1,184,621	61·4	134,963	7·0	1,794,194	93·0
**Stillbirth**	7,705	0·4	2,237	0·3	5,468	0·5	5,094	3·8	2,611	0·1
**Congenital anomalies**	42,336	2·2	38,364	5·2	3,972	0·3	3,975	2·9	38,361	2·1
**Multiples**	27,696	1·4	9,864	1·3	17,832	1·5	14,978	11·1	12,718	0·7
**Missing ACS information**	251,065	13·0	0	0	251,065	21·2	25,677	19·0	225,388	12·6
**Born < 28w or ≥ 42w GA**	75,102	3·9	36,676	4·9	38,426	3·2	7,835	5·8	67,267	3·7

Exclusion criteria as listed in rows of eTable 1.

Abbreviations: ACS = antenatal corticosteroids, n = number, w GA = weeks gestational age

© 2025 Decrue F et al. *JAMA Network Open*.

eTable 3. Descriptive characteristics according to term birth.

	Combined			Preterm			Term		
	n/mean	%/SD	missing information	n/mean	%/SD	missing information	n/mean	%/SD	missing information
**Total**	1,548,538	NA	0	86,482	NA	0	1,462,056	NA	0
**Antenatal corticosteroids**	49,263	3·18	0	34,806	40·25	0	14,457	0·99	0
**Preterm (28-36w GA)**	86,482	5·58	0	NA	NA	0	NA	NA	0
**Term (37-41w GA)**	1,462,056	94·42	0	NA	NA	0	NA	NA	0
**Maternal age, y**	29·4	5·7	<5	29·60	6·0	<5	29·4	5·7	<5
**Maternal BMI**	25·2	5·3	299,061	25·50	5·7	19,251	25·2	5·3	279,810
**Parity**			5,761			452			5,309
**Parity 0**	653,672	42·2	NA	41,424	47·9	NA	612,248	41·9	NA
**Parity 1**	537,323	34·7	NA	24,436	28·3	NA	512,887	35·1	NA
**Parity 2**	22,117	14·3	NA	11,401	13·2	NA	209,776	14·4	NA
**Parity ≥3**	124,844	8·1	NA	8,769	10·1	NA	121,836	8·3	NA
**Smokers**	278,762	18·0	56,678	19,949	23·07	4,576	258,813	17·7	52,102
**Any diabetes**	98,177	6·34	105,920	9,080	10·5	6,607	89,098	6·09	99,313
**GDM**	83,513	5·39	858,065	5,444	6·29	49,030	78,069	5·34	809,035
**Hypertension**	8,499	0·55	864,421	1,130	1·31	49,494	7,369	0·50	814,927
**Preeclampsia**	62,275	4·02	838,511	9,759	11·28	45,703	52,516	3·59	792,808
**Caesarean section**	338,741	21·87	<5	37,143	42·95	0	301,598	20·63	<5
**Birthweight, g**	3,443·9	551·4	1,442	2,422·6	627·1	202	3,504·2	483·2	1,240
**Female**	759,082	49·02	95	39,586	45·77	11	719,496	49·21	84
**APGAR <7**	24,490	1·58	92,862	5,458	6·31	5,939	19,032	1·3	86,923
**NICU admission**	143,633	9·28	14,916	47,344	54·74	1,235	96,289	6·59	13,681
**Gestational age at birth, w**	39·2	1·7	0	34·5	2	0	39·5	1·1	0

Abbreviations: BMI = body mass index, g = grams, GDM = gestational diabetes mellitus, n = number, NICU admission = neonatal intensive care unit admission, SD = standard deviation, w = weeks, w GA = weeks gestational age, y = years

© 2025 Decrue F et al. *JAMA Network Open*.

eTable 4. Descriptive characteristics according to ACS exposure.

	ACS-exposed				ASC non-exposed			
	**Term**		**Preterm**		**Term**		**Preterm**	
	**n/mean**	**%/SD**	**n/mean**	**%/SD**	**n/mean**	**%/SD**		
**Total**	14,457	NA	34,806	NA	1,447,599	NA	51,676	NA
**Maternal age, y**	29·9	6·0	29·7	6·1	29·4	5·7	29·5	6·0
**Maternal BMI**	25·6	6·2	25·8	5·9	25·2	5·3	25·3	5·6
**Parity**								
**Parity 0**	5,230	36·2	16,945	48·7	607,018	41·9	24,479	47·4
**Parity 1**	5,120	35·4	9,565	27·5	507,767	35·1	14,871	28·8
**Parity 2**	2,443	16·9	4,540	13·0	207,333	14·3	6,861	13·3
**Parity ≥3**	1,664	11·5	3,756	10·8	125,481	8·7	5,465	10·6
**Smokers**	2,981	20·6	8,172	23·5	255,832	17·7	11,777	22·8
**Any diabetes**	1,822	12·6	3,565	10·2	87,275	6·0	5,515	10·7
**GDM**	1,329	9·2	1,901	5·5	76,740	5·3	3,543	6·9
**Hypertension**	205	1·4	530	1·5	7,164	0·5	600	1·2
**Preeclampsia**	817	5·7	4,417	12·7	51,699	3·6	5,342	10·3
**Caesarean section**	5,916	40·9	19,203	55·2	295,682	20·4	17,940	34·7
**Birthweight, g**	3,254	540	2124	621	3,507	482	2,623	546
**Female**	7,078	49·0	16,066	46·2	712,418	49·2	23,520	45·5
**APGAR <7**	280	1·9	2,848	8·2	18,752	1·3	2,610	5·1
**NICU admission**	1,920	13·3	25,770	74·0	94,369	6·5	21,574	41·7
**Gestational age at birth, w**	38·3	1·2	33·3	2·2	39·5	1·1	35·3	1·3

Abbreviations: BMI = body mass index, g = grams, GDM = gestational diabetes mellitus, n = number, NICU admission = neonatal intensive care unit admission, SD = standard deviation, w = weeks, w GA = weeks gestational age, y = years

© 2025 Decrue F et al. *JAMA Network Open*.

eTable 5. Median age at first infection and median age at end of follow-up.

	Age at infection [a]		Age at end of follow-up [a]	
	**Median**	**IQR**	**Median**	**IQR**
**Respiratory infections**				
Overall	494	215 – 1,070	1,964	644 – 3,717
28-31 GA	320	147 - 673	1,197	300 – 3,335
32-33 GA	345	132 - 778	1,502	398 – 3,673
34-36 GA	416	165 - 893	1,664	492 – 3,588
37-38 GA	455	189 - 982	1,772	554 – 3,643
39-41 GA	516	231 – 1,115	2,029	688 – 3,742
**Non-respiratory infections**				
Overall	567	245 – 1,281	2,431	1,027 – 3,938
28-31 GA	424	176 - 946	2,273	735 – 3,997
32-33 GA	443	170 - 1003	2,292	801 – 4,056
34-36 GA	500	208 – 1,119	2,271	866 – 3,909
37-38 GA	532	227 – 1,201	2,314	912 – 3,915
39-41 GA	586	256 – 1,321	2,468	1,074 – 3,944

[a] age in days
abbreviations: IQR = interquartile range

© 2025 Decrue F et al. *JAMA Network Open*.

eTable 6. Sensitivity analyses including maternal BMI or hypertension or preeclampsia diagnoses into full adjusted model.

	N of subjects	Full adj. HR*	95% CI	N of subjects	Full adj. HR[a] with BMI	95% CI	N of subjects	Full adj. HR[a] with hypertension	95% CI	n of subjects	Full adj. HR[a] with preeclampsia	95%CI
**Respiratory infections**												
28-31 GA	7,168	1·07	0·98 – 1·17	5,960	1·06	0·96 – 1·17	3,231	1·08	0·96 – 1·21	3,582	1·09	0·98 – 1·22
32-33 GA	9,406	1·05	0·97 – 1·13	7,933	1·04	0·96 – 1·13	4,536	1·02	0·94 – 1·12	4,910	1·03	0·94 – 1·13
34-36 GA	59,001	1·10	1·06 – 1·14	50,595	1·11	1·07 – 1·15	27,454	1·08	1·03 – 1·14	29,635	1·09	1·04 – 1·14
37-38 GA	256,535	1·19	1·15 – 1·24	223,748	1·19	1·14 – 1·24	121,053	1·19	1·13 – 1·25	126,543	1·19	1·13 – 1·25
39-41 GA	1,056,594	1·27	1·21 – 1·32	920,346	1·28	1·23 – 1·34	506,830	1·27	1·21 – 1·33	518,959	1·27	1·21 – 1·33
**Non-respiratory infections**												
28-31 GA	7,166	0·97	0·85 – 1·10	5,958	0·93	0·81 – 1·07	3,231	0·78	0·64 – 0·94	3,582	0·84	0·70 – 1·01
32-33 GA	9,406	0·99	0·88 – 1·11	7,933	1·01	0·89 – 1·14	4,536	0·94	0·80 – 1·10	4,910	0·93	0·80 – 1·09
34-36 GA	59,000	1·17	1·11 – 1·23	50,594	1·21	1·15 – 1·28	27,453	1·19	1·10 – 1·29	29,634	1·18	1·10 – 1·28
37-38 GA	256,526	1·23	1·16 – 1·30	223,739	1·21	1·15 – 1·29	121,044	1·23	1·13 – 1·34	126,534	1·23	1·13 – 1·34
39-41 GA	1,056,563	1·31	1·22 – 1·40	920,317	1·33	1·24 – 1·43	506,799	1·30	1·20 – 1·41	513,928	1·30	1·20 – 1·41

[a] Full adjusted model: adjusted for sex, year of birth, gestational age at birth, country, mode of delivery, maternal age, parity, maternal smoking, maternal diabetes, birthweight adjusted for gestational age and sex

abbreviations: adj. HR = adjusted hazard ratio, GA = gestational age in weeks, n = number, 95% CI = 95% confidence interval

© 2025 Decrue F et al. *JAMA Network Open*.

eTable 7. Sensitivity analyses performing stratified analyses by country.

	Scotland			Finland		
	**N of subjects**	**Full adj. HR[a]**	**95% CI**	**N of subjects**	**Full adj. HR[a]**	**95% CI**
**Respiratory infections**						
28-31 GA	4,526	1·06	0·92 – 1·22	2,642	1·10	0·97 – 1·23
32-33 GA	5,466	1·11	0·96 – 1·28	3,940	1·02	0·93 – 1·12
34-36 GA	34,046	1·12	1·06– 1·18	24,955	1·08	1·02 – 1·13
37-38 GA	140,322	1·19	1·12 – 1·27	116,213	1·18	1·12 – 1·25
39-41 GA	562,810	1·30	1·16 – 1·46	493,784	1·27	1·21 – 1·33
**Non-respiratory infections**						
28-31 GA	4,524	1·13	0·96 – 1·34	2,642	0·76	0·61 – 0·94
32-33 GA	5,466	1·04	0·88 – 1·22	3,940	0·99	0·84 – 1·18
34-36 GA	34,046	1·15	1·08 – 1·22	24,954	1·22	1·11 – 1·34
37-38 GA	140,323	1·23	1·15 – 1·32	116,203	1·20	1·10 – 1·32
39-41 GA	562,810	1·45	1·29 – 1·63	493,753	1·29	1·19 – 1·41

[a] Full adjusted model: adjusted for sex, year of birth, gestational age at birth, mode of delivery, maternal age, parity, maternal smoking, maternal diabetes, birthweight adjusted for gestational age and sex
abbreviations: adj. HR = adjusted hazard ratio, GA = gestational age in weeks, n = number, 95% CI = 95% confidence interval

© 2025 Decrue F et al. *JAMA Network Open*.

eTable 8. Sensitivity analyses analysing the association of exposure to antenatal corticosteroids and infectious diseases throughout different ages of childhood and adolescence.

	1st year				2nd to 4th year				4th year to end of follow-up			
	N of subjects	N cases	Full adj. HR[a]	95% CI	N of subjects	N cases	Full adj. HR[a]	95% CI	N of subjects	N cases	Full adj. HR[a]	95% CI
**Respiratory infections**												
overall	220,362	149,833	1·06	1·03 – 1·09	385,378	170,044	1·04	1·01 - 1·07	782,710	61,630	1·04	0·98 - 1·10
28-31 GA	2,151	1,851	1·08	0·95– 1·22	1,896	1,215	1·09	0·93 – 1·28	3,121	284	0·96	0·71 – 1·28
32-33 GA	2,295	1,879	1·12	1·01 – 1·25	2,609	1,367	0·97	0·86 – 1·09	4,500	385	0·88	0·70 – 1·10
34-36 GA	11,894	8,817	1·00	0·95 – 1·06	16,829	7,974	1·04	0·98 – 1·10	30,262	2,526	1·04	0·94 – 1·16
37-38 GA	46,775	32,551	1·00	0·95 – 1·06	73,699	32,784	0·96	0·90 – 1·02	136,007	11,123	1·05	0·92 – 1·20
39-41 GA	157,247	104,735	1·14	1·07 – 1·21	290,345	126,704	1·01	0·94 – 1·08	608,820	47,312	1·09	0·96 – 1·24
**Non-respiratory infections**												
overall	141,412	66,543	1·05	1·01 – 1·09	336,518	81,788	0·99	0·95 – 1·03	910,731	37,742	1·09	1·02 - 1·17
28-31 GA	1,103	751	1·02	0·83 – 1·25	1,740	697	0·94	0·77 – 1·16	4,323	245	0·93	0·68 – 1·28
32-33 GA	1,269	789	1·04	0·86 – 1·25	2,367	743	0·94	0·78 – 1·13	5,770	280	0·87	0·66 – 1·14
34-36 GA	7,170	3,821	1·00	0·92 – 1·08	15,151	4,184	0·98	0·91 – 1·06	36,679	1,709	1·17	1·04 – 1·33
37-38 GA	29,598	14,317	0·98	0·90 – 1·06	65,537	16,357	0·97	0·89 – 1·06	161,391	7,157	1·26	1·08 – 1·47
39-41 GA	102,272	46,865	1·15	1·03 – 1·28	251,723	59,807	0·93	0·83 – 1·03	702,568	28,351	1·18	1·01 – 1·39

[a] Full adjusted model: adjusted for sex, year of birth, gestational age at birth, mode of delivery, maternal age, parity, maternal smoking, maternal diabetes, birthweight adjusted for gestational age and sex
abbreviations: adj. HR = adjusted hazard ratio, GA = gestational age in weeks, n = number, 95% CI = 95% confidence interval

© 2025 Decrue F et al. *JAMA Network Open*.

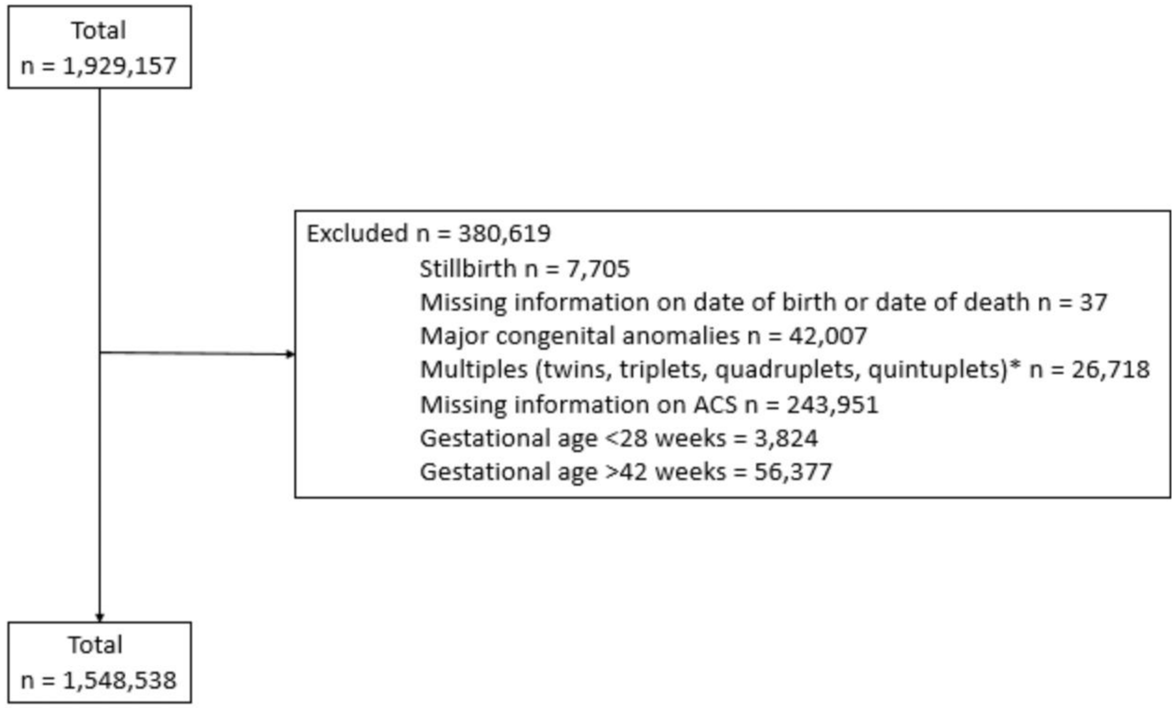

eFigure 1. Flowchart of exclusion criteria.
Abbreviations: ACS = antenatal corticosteroids

© 2025 Decrue F et al. *JAMA Network Open*.

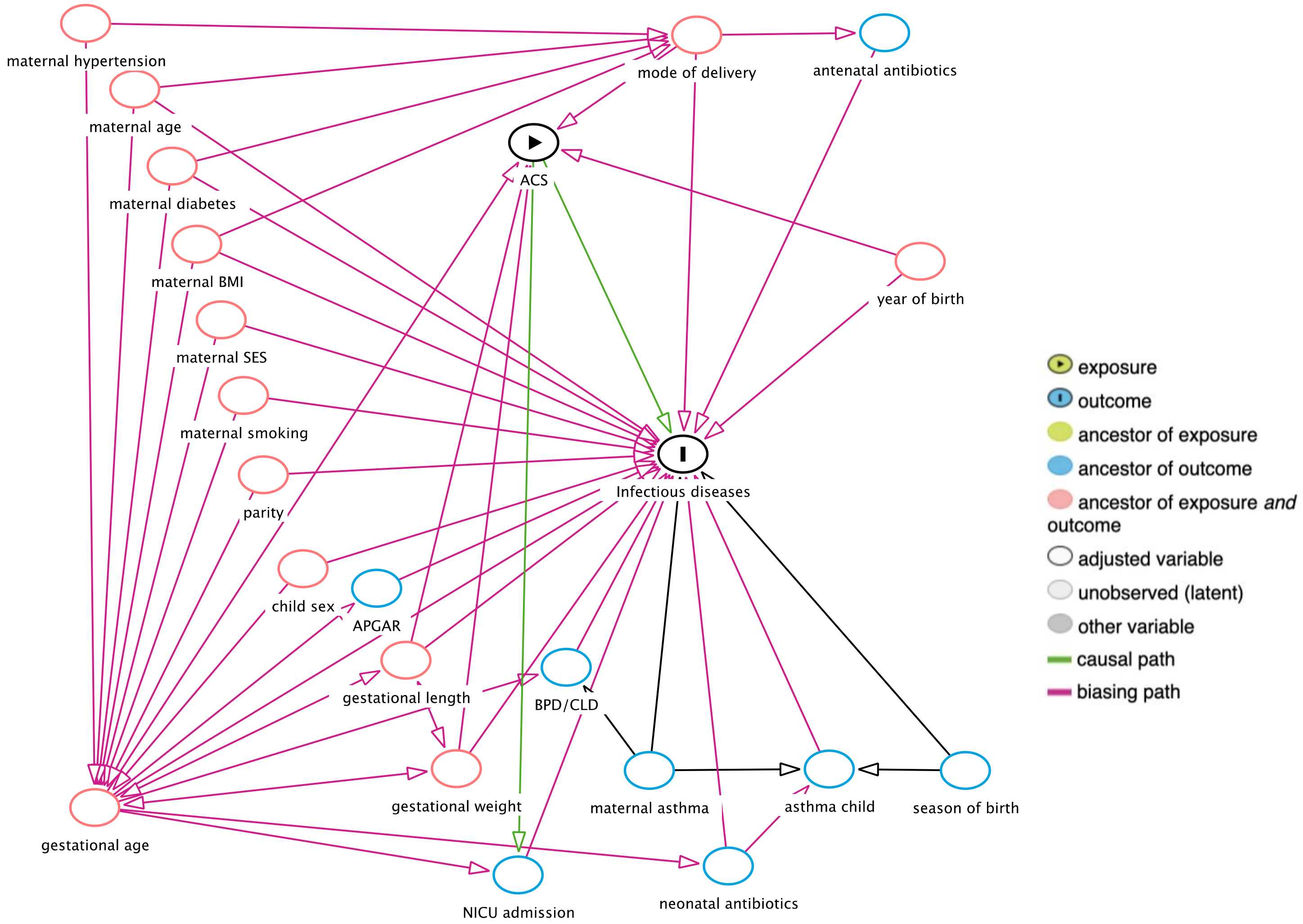

eFigure 2. Complex direct acyclic graph (DAG)
Abbreviations: ACS = antenatal corticosteroids, BPD/CLD = bronchopulmonary dysplasia / chronic lung disease of infancy, BMI = body mass index, SES = socioeconomic status, NICU admission= neonatal intensive care unit admission.

© 2025 Decrue F et al. *JAMA Network Open*.

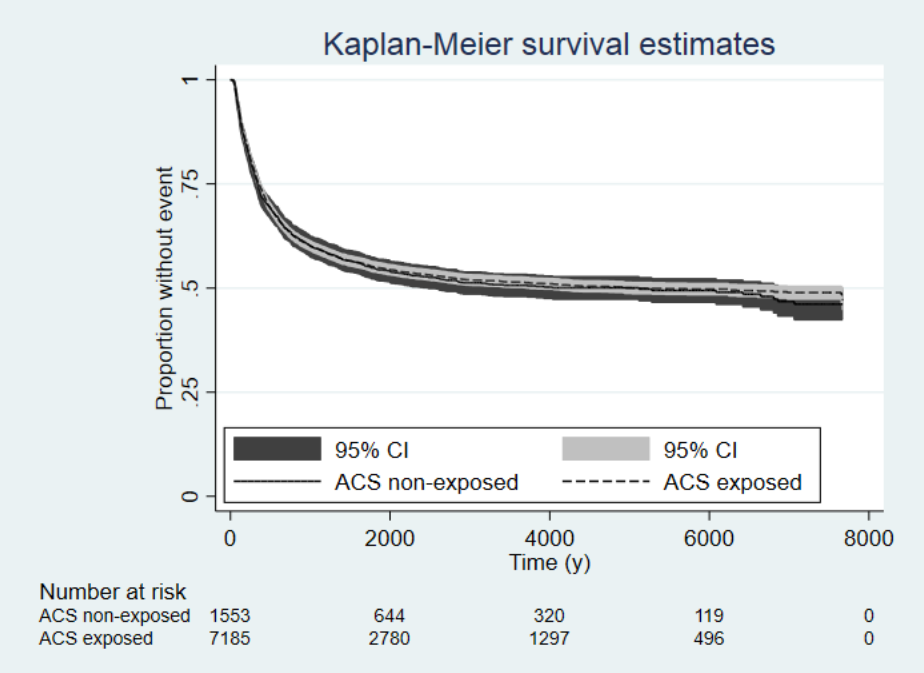

eFigure 3. Kaplan-Meier curve of respiratory infections for preterm children born 28^{+0} – 31^{+6} weeks gestation

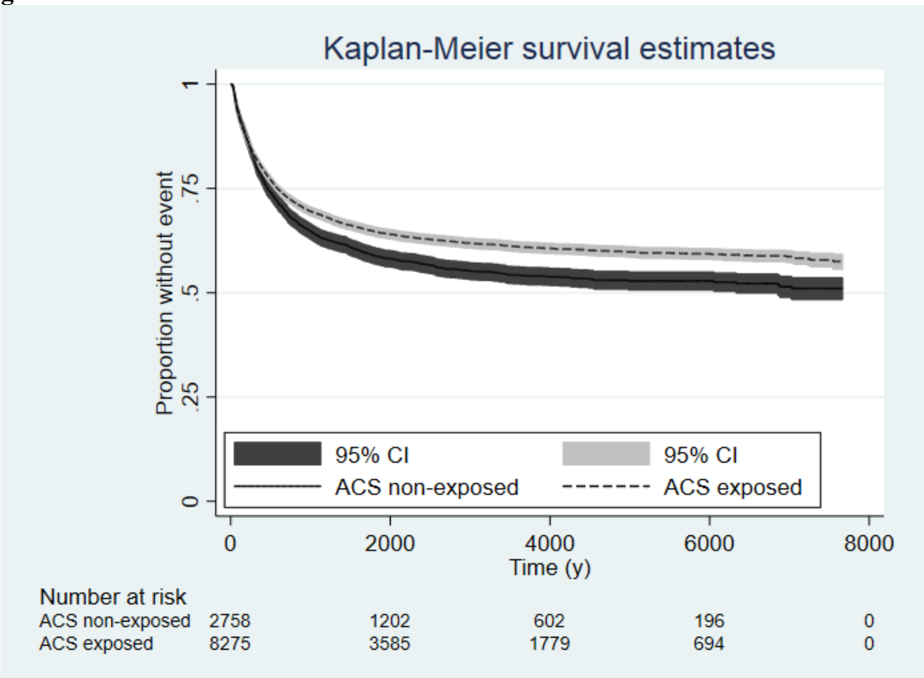

eFigure 4. Kaplan-Meier curve of respiratory infections for preterm children born 32^{+0} – 33^{+6} weeks gestation

© 2025 Decrue F et al. *JAMA Network Open*.

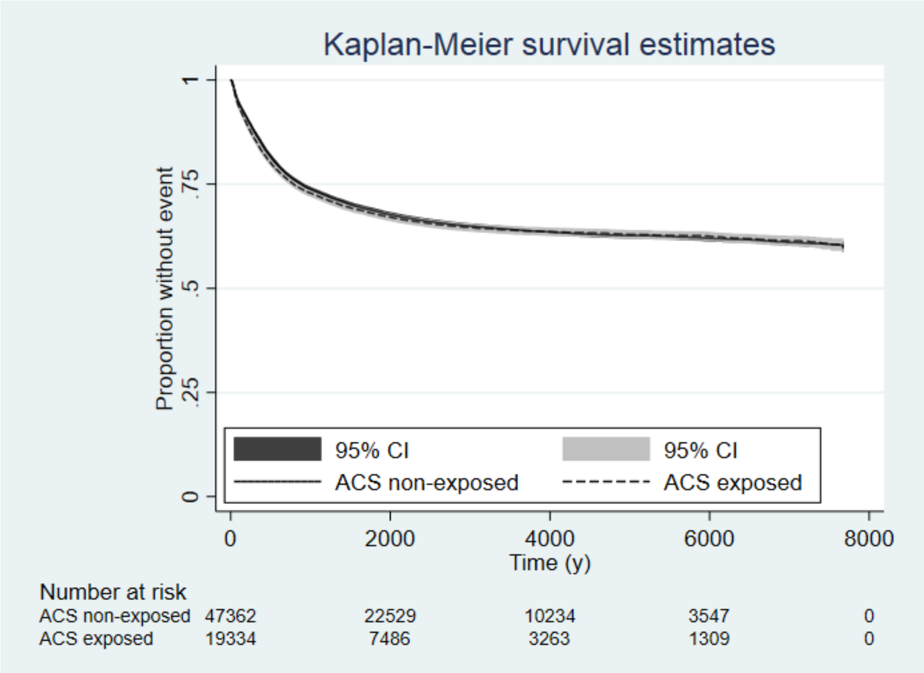

eFigure 5. Kaplan-Meier curve of respiratory infections for preterm children born 34^{+0} – 36^{+6} weeks gestation

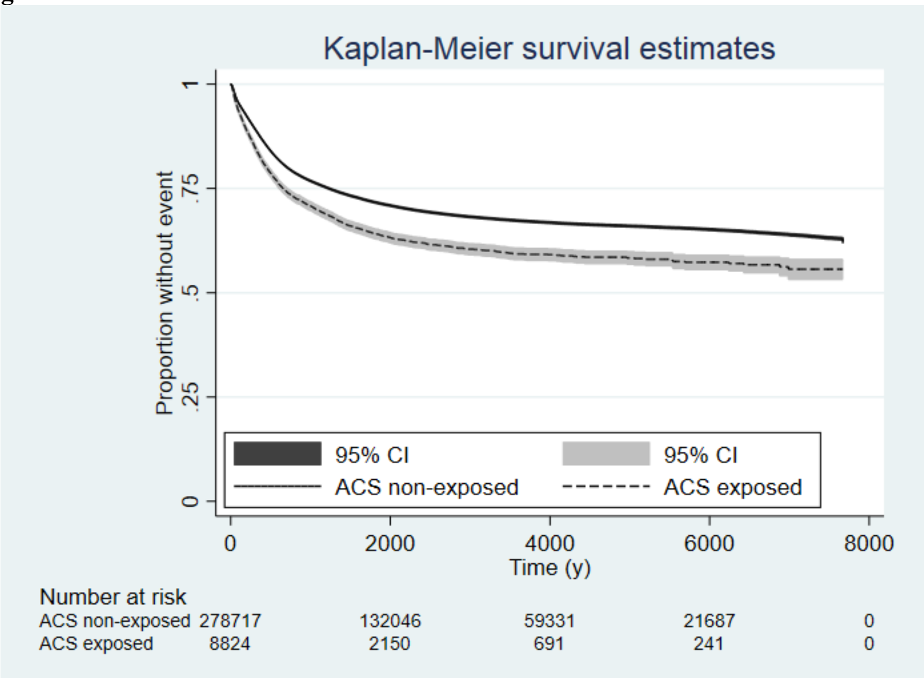

eFigure 6. Kaplan-Meier curve of respiratory infections for preterm children born 37^{+0} – 38^{+6} weeks gestation

© 2025 Decrue F et al. *JAMA Network Open*.

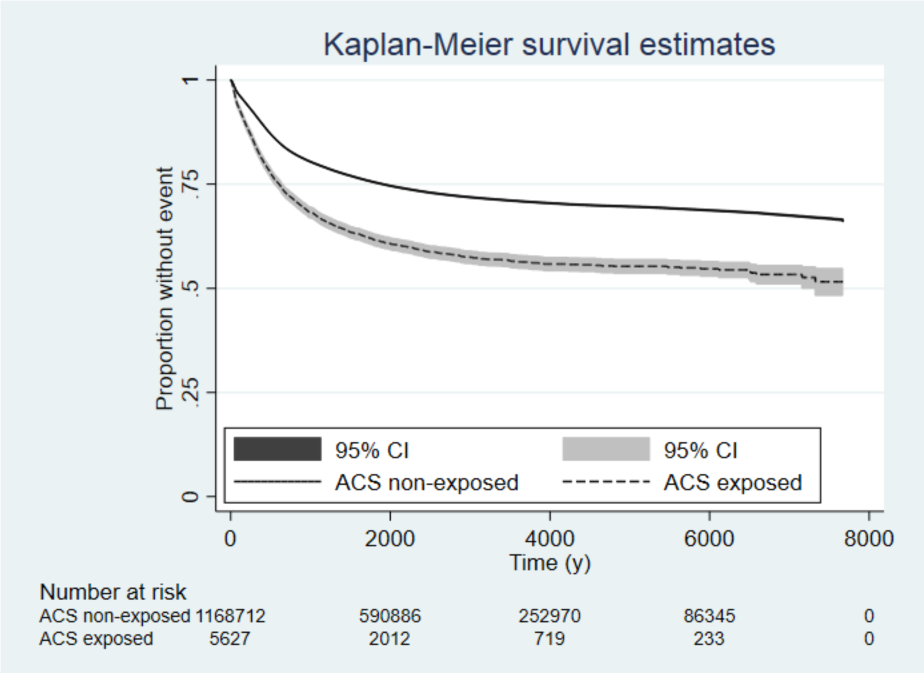

eFigure 7. Kaplan-Meier curve of respiratory infections for term children born 39^{+0} – 41^{+6} weeks gestation

© 2025 Decrue F et al. *JAMA Network Open*.

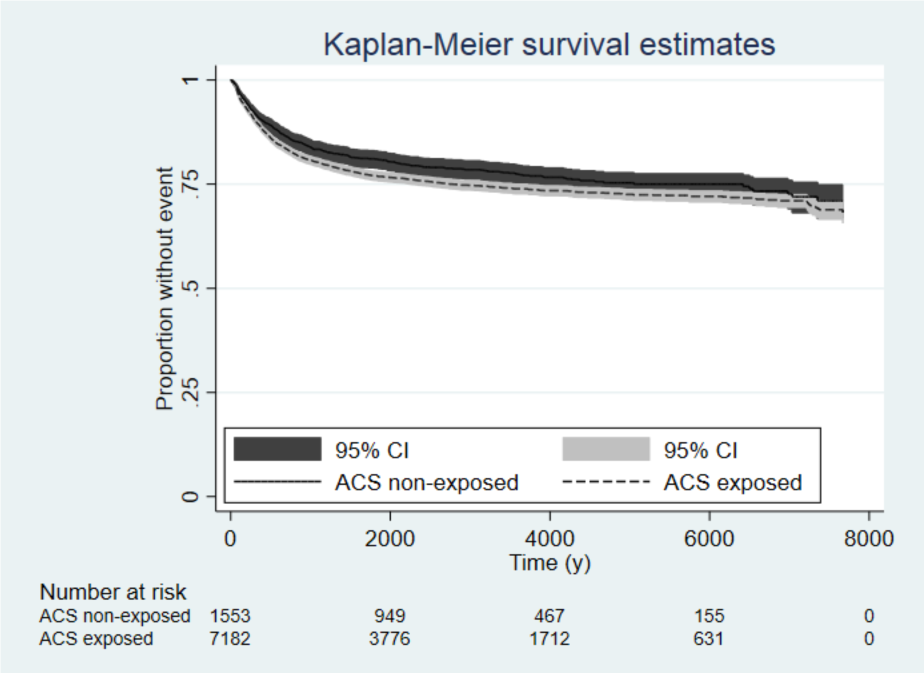

eFigure 8. Kaplan-Meier curves of non-respiratory infections for preterm children born 28^{+0} – 31^{+6} weeks gestation

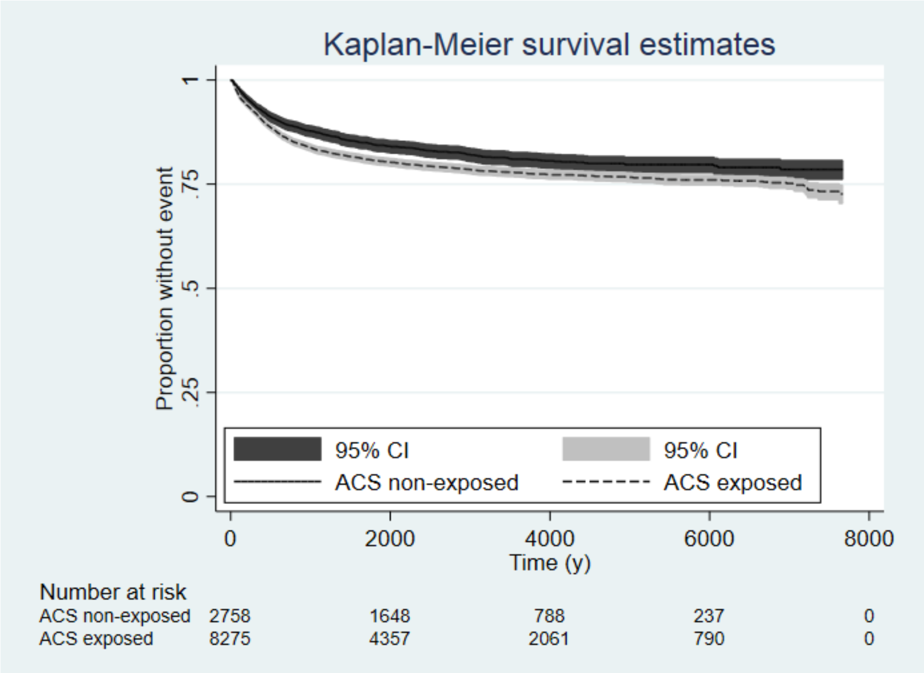

eFigure 9. Kaplan-Meier curves of non-respiratory infections for preterm children born 32^{+0} – 33^{+6} weeks gestation

© 2025 Decrue F et al. *JAMA Network Open*.

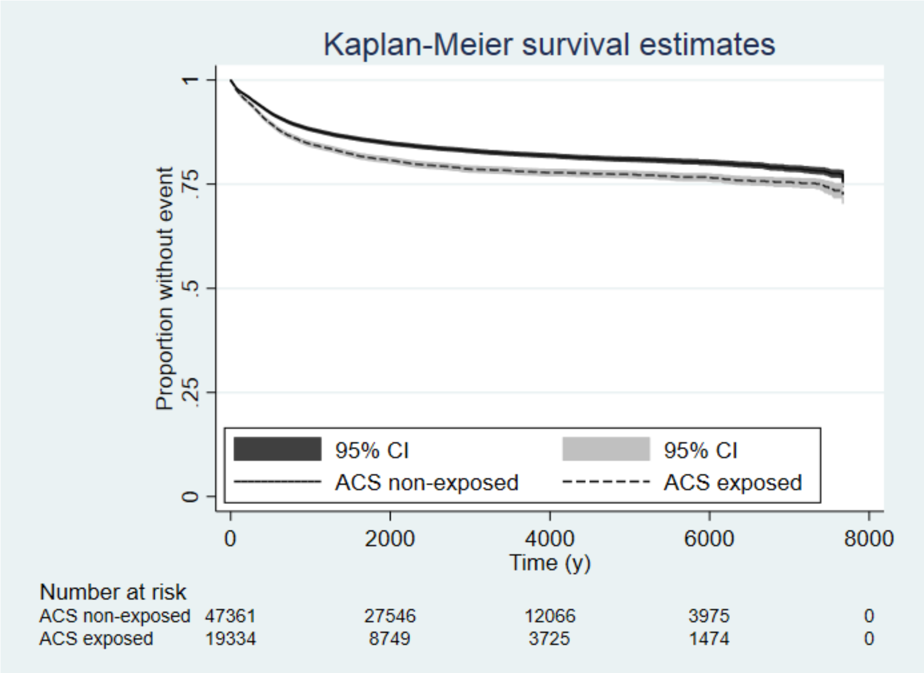

eFigure 10. Kaplan-Meier curves of non-respiratory infections for preterm children born 34^{+0} – 36^{+}6 weeks gestation

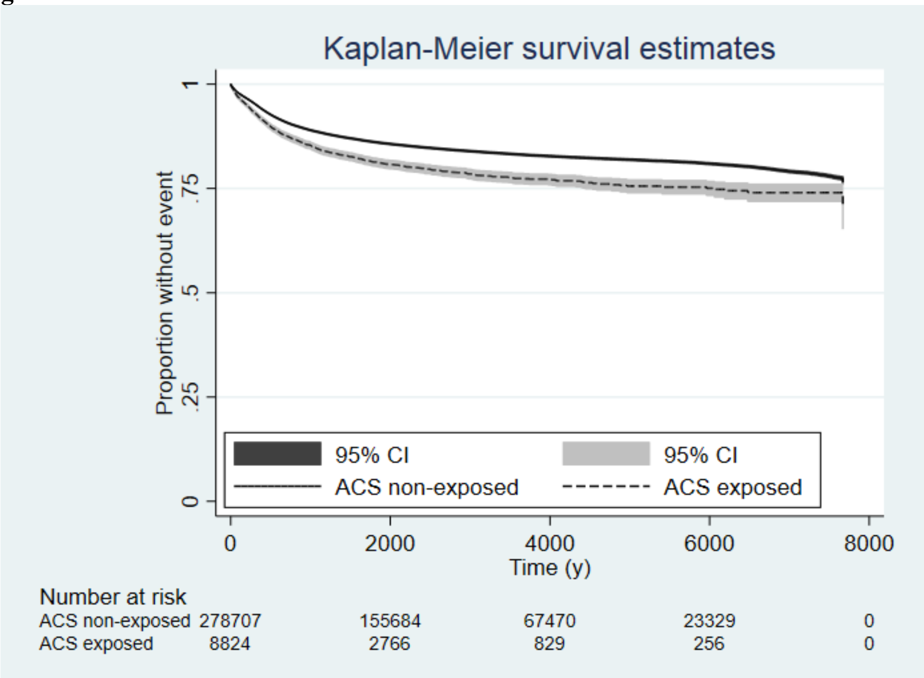

eFigure 11. Kaplan-Meier curves of non-respiratory infections for preterm children born 37^{+0} – 38^{+6} weeks gestation

© 2025 Decrue F et al. *JAMA Network Open*.

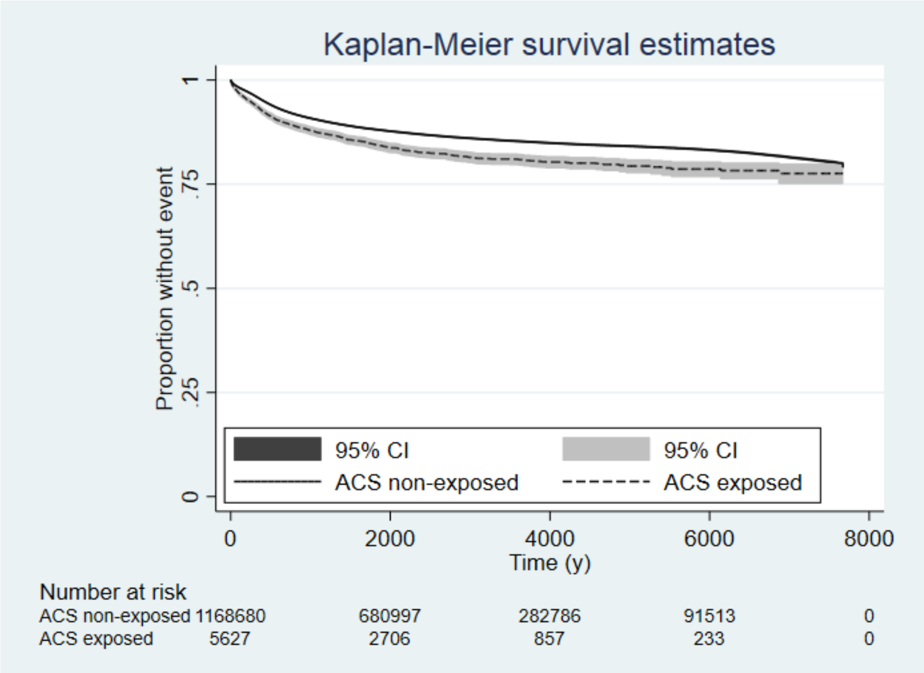

eFigure 12. Kaplan-Meier curves of non-respiratory infections for preterm children born 39^{+0} – 41^{+6} weeks gestation

© 2025 Decrue F et al. *JAMA Network Open*.
