# Supplementary material for: Antenatal Corticosteroids and Infectious Diseases Throughout Childhood
Source: JAMA Netw Open. 2025 Oct 13;8(10):e2536809. doi: 10.1001/jamanetworkopen.2025.36809 (PMC12519305; doi:10.1001/jamanetworkopen.2025.36809)
Supplement: Supplement 2. — Consortium for the Study of Pregnancy Treatments (Co-OPT) Investigators [file jamanetwopen-e2536809-s002.pdf]

Supplemental Online Content: Nonauthor Collaborators
*First name, last name, and suffix (if applicable) are required and will appear in PubMed.

*Group Name(s): Consortium for the Study of Pregnancy Treatments (Co-OPT) Investigators								
*First Name and Middle Initial(s)	*Last Name	*Suffix (eg, Jr, III)	Academic Degrees	Institution	Location (city, state/province, country)	Role or Contribution, eg, chair, principal investigator	Group (if more than 1 Group listed in the byline) and/or Subgroup (eg, Steering Committee)	
Abigail	Fraser		Prof.	University of Bristol, Bristol Medical School	Clifton, Bristol, UK			abigail.fraser@bristol.ac.uk
Alison	McNulty			The Premature Baby Charity for Northern Irland				alison@tinylife.org.uk
Ashraf	Nabhan		Prof.	Department of Obstetrics and Gynecology, Ain Shams University	Cairo, Egypt			anabhan@med.asu.edu.eg
Aziz	Sheikh			Usher Institute, College of Medicine and Veterinary Medicine / Nuffield Department of Primary Care Health Sciences, University of Oxford, Oxford, England	Edinburgh, UK / Oxford, England			aziz.sheikh@ed.ac.uk
Ben	Mol		Prof.	Department of Obstetrics and Gynecology, Monash University, Melbourne, Australia	Melbourne, Victoria, Australia			ben.mol@monash.edu
Christy	Woolcott	G.	PhD	Department of Pediatrics, Department of Community Health and Epidemiology, Department of Obstetrics and Gynecology, Dalhousie University, Halifax, Canada	Halifax, Canada			Christy.Woolcott@iwk.nshealth.ca
Cynthia	Gyamfi Bannerman		MD	Department of Obstetrics, Gynecology and reproductive sciences, Columbia University of California	San Diego, California, US			cg2231@cumc.columbia.edu
Devender	Roberts		MD	Liverpool Women's NHS Foundation Trust, Liverpool, UK	Liverpool, UK			devender.roberts@lwh.nhs.uk
Ewoud	Schuit		PhD	Julius Center for Health Sciences and	Utrecht, Netherlands			eschuit@umcutrecht.nl
Stefan	Kuhle		PhD	Institute for Medical Biostatistics, Epi	Mainz, Germany			stefan.kuhle@uni-mainz.de
Eyal	Krispin		MD	Division of Fetal Medicine and Surger	Boston, USA			eyalkrispin@gmail.com
Jane	Norman		Prof.	University of Nottingham, Nottinghai	Nottingham, UK			ci19881@bristol.ac.uk
Jeeva	John			School of Primary Care, Population S	Southhampton, UK			jeeva.john@ed.ac.uk;
John	Wright		Prof.	Kingsbury Hospital, Cape Town, Sout	Cape Town, South Africa			john.wright@bthft.nhs.uk
Karel	Allegaert		Prof.	Department of Pharmaceutical and P	Leuven, Belgium			allegaertkarel@hotmail.com
Kristjana	Einarsdottir		MD	Centre of Public Health Sciences, Fac	Reykjavik, Iceland			ke@hi.is

Supplemental Online Content: Nonauthor Collaborators
*First name, last name, and suffix (if applicable) are required and will appear in PubMed.

***First Name and Middle Initial(s)**	***Last Name**	***Suffix (eg, Jr, III)**	Academic Degrees	Institution	Location (city, state/province, country)	Role or Contribution, eg, chair, principal investigator	Group (if more than 1 Group listed in the byline) and/or Subgroup (eg, Steering Committee)	
Lani	Florian							lani.florian@ed.ac.uk
Lars	Henning Pedersen		Prof.	Department of Clinical Medicine, Aar	Aarhus, Denmark			lhp@clin.au.dk
Mandy	Daly			Education and Research, Irish Neonat	Wicklow, Ireland			mandy.daly@yahoo.co.uk
Mika	Gissler			Department of Data and Analytics, Fi	Helsinki, Finland / Stockholm, Sweden			mika.gissler@thl.fi
Rachael	Wood		Prof.	Public Health Scotland, Edinburgh, Sc	Edinburgh, Scotland			Rachael.Wood@phs.scot
Richard	Riley		Prof.	Department of Applied Health Sciences, University of Birmingham	Birmingham, UK			r.d.riley@bham.ac.uk
Sarah	Murray		PhD	MRC Centre for Reproductive Health,	Edinburgh, Scotland, UK			sarah.murray@ed.ac.uk
Sohinee	Bhattacharya		PhD	Aberdeen Centre for Women's Healtl	Aberdeen, Scotland, UK			sohinee.bhattacharya@abdn.ac.uk
Ting	Shi		PhD	Usher Institute, The University of Edi	Edinburgh, Scotland, UK			ting.shi@ed.ac.uk
Joanne	Blair-Gray			Usher Institute, The University of Edi	Edinburgh, Scotland, UK			Joanne.Blair@ed.ac.uk
Emma	McGoldrick		MD	Family Health Division, Liverpool Wo	Liverpool, UK			emmalouise.mcgoldrick@lwh.nhs.uk
