# Supplementary material for: Antenatal Corticosteroids and Infectious Diseases Throughout Childhood
Source: JAMA Netw Open. 2025 Oct 13;8(10):e2536809. doi: 10.1001/jamanetworkopen.2025.36809 (PMC12519305; doi:10.1001/jamanetworkopen.2025.36809)
Supplement: Supplement 3. — Data Sharing Statement [file jamanetwopen-e2536809-s003.pdf]

Data Sharing Statement

Decrue. Antenatal Corticosteroids and Infectious Diseases Throughout Childhood. *JAMA Netw Open*. Published October 13, 2025. doi:10.1001/jamanetworkopen.2025.36809

Data
Data available: No

Additional Information
Explanation for why data not available: The data used for this study was secondary data, which was hosted by Public Health Scotland. In accordance with the Finnish data protection laws, individual participant data cannot be made publicly available. To access deidentified data with permission from the register authority proposals have to be directed to the Finnish Social and Health Data Permit Authority Findata (findata.fi/en/) and for the Scottish data to the Research Data Scotland (https://www.researchdata.scot/). To gain access to the data, the researchers will need to attest to and sign a data-access agreement.
